# Exploring the Structure–Activity Relationships of Albumin-Targeted Picoplatin-Based Platinum(IV) Prodrugs

**DOI:** 10.1021/acs.inorgchem.4c05269

**Published:** 2025-01-29

**Authors:** Martijn Dijkstra, Hemma Schueffl, Barbora Adamova, Oliver Baumfried, Alexander Kastner, Walter Berger, Bernhard K. Keppler, Petra Heffeter, Christian R. Kowol

**Affiliations:** † Faculty of Chemistry, Institute of Inorganic Chemistry, University of Vienna, Waehringer Str. 42, 1090 Vienna, Austria; ‡ Vienna Doctoral School in Chemistry (DoSChem), University of Vienna, Waehringer Str. 42, 1090 Vienna, Austria; § Center for Cancer Research and Comprehensive Cancer Center, Medical University of Vienna, Borschkegasse 8a, 1090 Vienna, Austria; ∥ Research Cluster “Translational Cancer Therapy Research”, 1090 Vienna, Austria

## Abstract

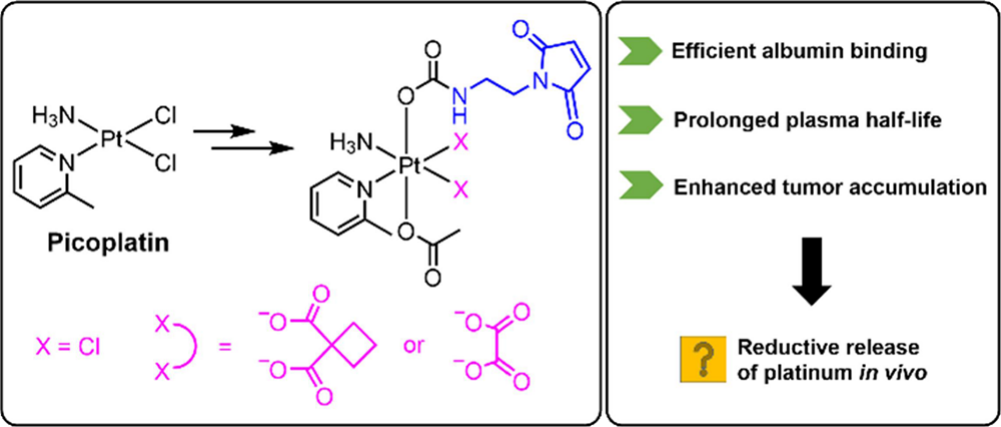

Platinum(II) complexes prevail as first-line treatment for many cancers but are associated with serious side effects and resistance development. Picoplatin emerged as a promising alternative to circumvent GSH-induced tumor resistance by introducing a bulky 2-picoline ligand. Although clinical studies were encouraging, picoplatin did not receive approval. Interestingly, the anticancer potential of prodrugs based on picoplatin is widely underexplored, and even less so the respective tumor-targeting approaches. We synthesized two new “hybrid” picoplatin(II) derivatives with an oxalate or cyclobutane dicarboxylate leaving group and their corresponding platinum(IV) prodrugs with an albumin-targeting maleimide moiety or a succinimide as reference. Picoplatin(II) and its derivatives indeed reacted much slower with GSH compared to the respective analogs cisplatin, carboplatin, or oxaliplatin. While PicoCarbo(IV) and PicoOxali(IV) were reduced slowly in the presence of ascorbic acid, picoplatin(IV) was extremely unstable. All three prodrugs were widely inactive in the MTT assays. The platinum(IV)-maleimide complexes rapidly bound to albumin with stable conjugates for >25 h. Albumin-binding resulted in elevated platinum plasma levels, prolonged blood circulation, and enhanced tumor accumulation of the prodrugs in mice bearing CT26 tumors. However, only maleimide-functionalized PicoCarbo(IV) and picoplatin(II) significantly inhibited tumor growth. One possible explanation is that for albumin-binding platinum(IV) prodrugs, the bulky 2-picoline moiety prevents sufficient activation/reduction to unlock their full anticancer potential.

## Abstract 2

Picoplatin was developed to counteract tumor resistance but faced limited clinical success. In this report, novel albumin-targeted platinum(IV)-maleimide prodrugs based on picoplatin demonstrated enhanced plasma stability and tumor accumulation in mice. However, despite reduced GSH reactivity and prolonged circulation, only one derivative effectively inhibited tumor growth. The bulky 2-picoline moiety likely impedes sufficient prodrug activation. This study highlights challenges in optimizing platinum(IV)-based prodrug design for improved anticancer drugs.

## Introduction

The platinum(II) complexes cisplatin, carboplatin, and oxaliplatin remain a cornerstone of cancer treatment and are involved in approximately 50% of all chemotherapy regimens.^1,2^ However, severe side effects of platinum(II) chemotherapy, due to the lack of selectivity for cancerous cells over healthy tissue and intrinsic resistance development, strongly limit their clinical application.^3,4^ Platinum(II) resistance primarily involves factors that affect the formation of platinum-DNA adducts, reduce drug uptake, and alter the induced apoptotic pathways.^5,6^ In particular, elevated levels of glutathione (GSH), a paramount cellular antioxidant and detoxifier,^7^ are frequently observed in cisplatin resistance.^8−10^ Increased intracellular concentrations of GSH rapidly deactivate cisplatin by formation of a binary [Pt(SG)_2_(NH_3_)_2_] complex^11^ and, therefore, efficiently suppress its therapeutic effect.^12^ The 2-picoline-containing picoplatin (also commonly termed AMD473) was rationally designed as a “sterically hindered” cisplatin, a property that would shield the platinum(II) core from interaction with (and thus deactivation by) biological nucleophiles such as GSH or metallothionein.^13^ In fact, in vitro studies showed that picoplatin retained its anticancer activity in the presence of increased GSH levels^14^ and exhibited superior antitumor activity against cisplatin-resistant human ovarian xenograft models compared to cisplatin and carboplatin.^15,16^ Based on these encouraging preclinical results, picoplatin advanced into clinical studies.^17^ Although reasonable tolerability was confirmed in various phase I trials (with myelosuppression as dose-limiting factor), picoplatin failed to show improved overall survival as first-line and second-line treatment for patients with non-small cell lung cancer in a phase III trial.^18−20^ In addition, picoplatin was examined in phase II either in combination with 5-fluorouracil and leucovorin against colorectal cancer^21,22^ or with docetaxel and prednisone against prostate cancer.^23^ Several attempts have been made to improve the properties of picoplatin, e.g., via the introduction of different pyridinecarboxylates as leaving group ligands,^24^ formation of dinuclear complexes,^25^ or encapsulation in nanomaterials.^26,27^ Interestingly, although the scientific interest in the last years strongly shifted toward the development of platinum(IV) prodrugs, due to their improved kinetic inertness and higher degree of functionalization,^28−30^ only a handful of platinum(IV) analogues based on picoplatin have been reported.^31−35^ The earliest example from Hambley and coworkers comprises the preparation and characterization of the picoplatin(IV)-dihydroxido complex.^31^ The authors highlighted the steric clash between the methyl group of 2-picoline and the axial hydroxido ligand, which was supported by crystallographic and electrochemical investigations. Osella and coworkers reported fast reduction (average *t*_1/2_ of 30 min) of a series of picoplatin(IV)-dicarboxylato complexes in the presence of 5 equiv GSH at 37 °C.^34^ Some of these picoplatin(IV) derivatives were several-fold more active in MM98R cell lines in comparison to cisplatin. Additional attempts have been reported to improve the anticancer activity of picoplatin(IV) complexes against several cisplatin-resistant cell lines via conjugation to targeting peptides^33^ or via liposome encapsulation.^35^

Maleimides are well-known for their selective conjugation to cysteine-34 of endogenous albumin, which in turn enhances the stability and half-life of conjugated drugs^36^ and increases their intratumoral accumulation via the enhanced permeability and retention (EPR) effect.^37^ Furthermore, there is increasing evidence that cancer cells *per se* have a high albumin uptake to cover their enhanced need for nutrients and amino acids.^38^ A famous example is aldoxorubicin, a maleimide derivative of doxorubicin, which received orphan drug designation for the treatment of soft tissue sarcoma.^39^ In the past decade, our group reported several platinum(IV)-maleimido complexes with distinctly improved antitumor activity compared to cisplatin and oxaliplatin.^40−42^ Envisioned by our previous efforts and results, we synthesized a panel of novel platinum(II) complexes based on picoplatin as well as their succinimide- and maleimide-bearing platinum(IV) derivatives. First, the interactions of the platinum(II) cores with GSH were investigated under physiological conditions in comparison to cisplatin, carboplatin, and oxaliplatin to confirm the impact of steric hindrance exerted by 2-picoline. Then, the reduction kinetics of the platinum(IV)-succinimide complexes were studied as well as the short-term (72 h) and long-term (7 days) cytotoxicity in vitro via MTT and colony formation assays, respectively. Finally, albumin binding, serum pharmacokinetics, organ distribution, and anticancer activity of platinum(IV)-maleimide complexes were investigated in CT-26 tumor-bearing Balb/c mice.

## Results and Discussion

### Synthesis of Novel Picoplatin(II) Analogues and Their Platinum(IV) Derivatives

**Scheme 1:**
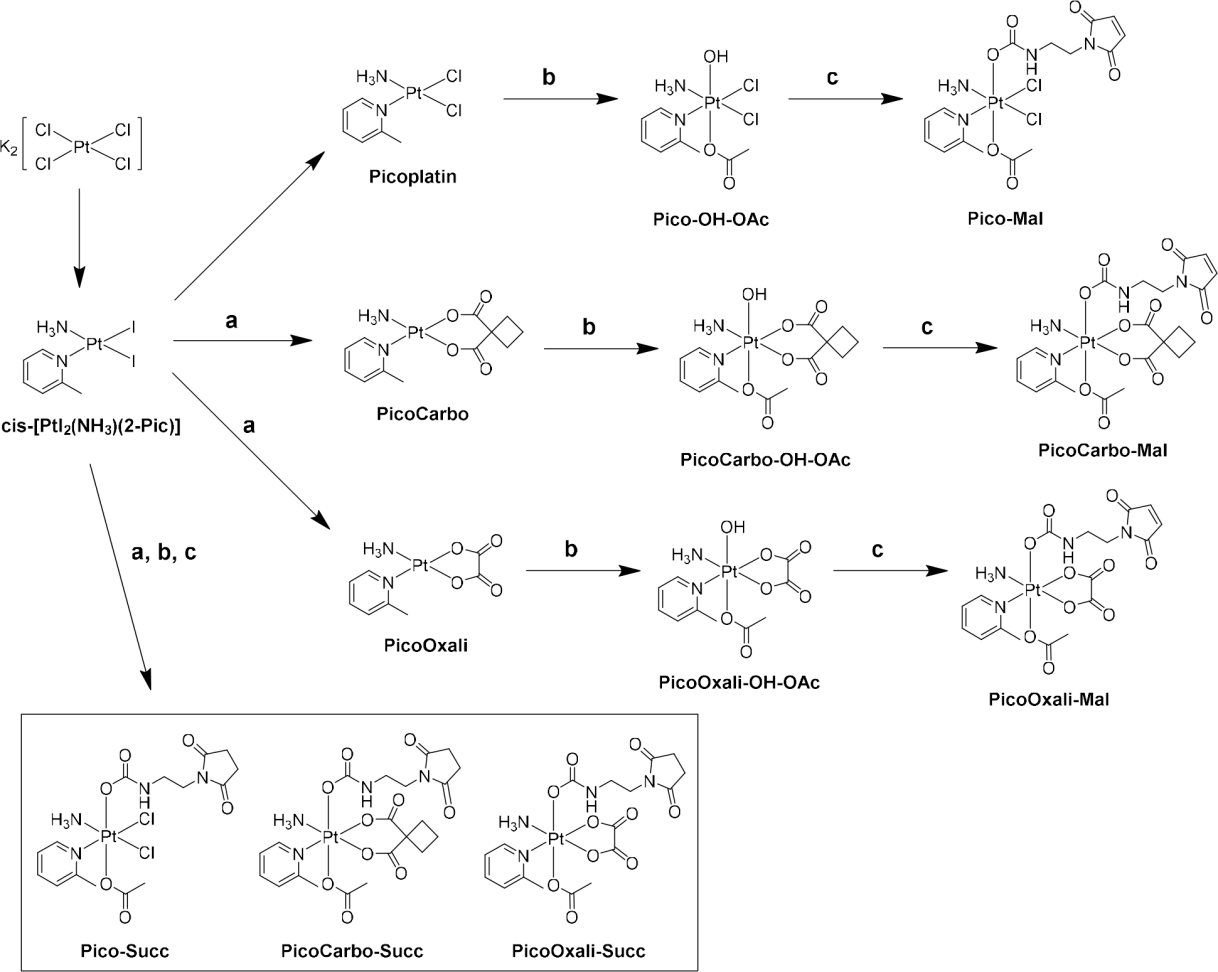
Synthetic Route for the Platinum(IV)-maleimide and Platinum(IV)-Succinimide Complexes (a) Silver cyclobutyldicarboxylate or silver oxalate, MQ-H_2_O, room temperature (RT); (b) H_2_O_2_ (50 wt % solution in H_2_O), acetic acid (AcOH), RT; (c) 1-(2-isocyanatoethyl)pyrrolidine-2,5-dione or 1-(2-isocyanatoethyl)-1*H*-pyrrole-2,5-dione, dimethylformamide (DMF), RT.

Picoplatin was synthesized, as previously described by Hambley and coworkers.^31^ To study the impact of introducing different leaving groups, novel picoplatin(II) analogs were prepared. Two “hybrid” complexes were designed by exchanging the chlorido ligands from picoplatin with the dicarboxylato ligands from carboplatin (cyclobutyl dicarboxylate) and oxaliplatin (oxalate), respectively. Therefore, the intermediate *cis*-[PtI_2_(NH_3_)(2-Pic)] was reacted with the silver salts of the respective dicarboxylic acids^43^ to obtain **PicoCarbo** and **PicoOxali** (Scheme 1). Subsequently, the platinum(II) complexes were oxidized asymmetrically with hydrogen peroxide in acetic acid to generate the platinum(IV)-acetato precursor complexes Pico-OH-OAc, PicoCarbo-OH-OAc, and PicoOxali-OH-OAc.^44^ The final platinum(IV) complexes were synthesized according to a general procedure reported by our group.^41^ In more detail, the isocyanate derived from commercially available 3-maleimidopropionic acid was reacted with each of the platinum(IV)-OH-OAc species to yield **Pico-Mal**, **PicoCarbo-Mal**, and **PicoOxali-Mal**. Due to the only moderate stability of the maleimide moiety at physiological conditions,^45^ the respective platinum(IV)-succinimide derivatives were additionally synthesized in a similar fashion. To that end, the isocyanate derived from 3-succinatopropionic acid was used to afford **Pico-Succ**, **PicoCarbo-Succ**, and **PicoOxali-Succ**. These complexes could also be used as references, as they are unable to bind to thiol groups. Interestingly, all PicoCarbo(IV) derivatives were obtained as a mixture of two isomers. Isomer interconversion of **PicoCarbo-Succ** was confirmed by ^1^H nuclear magnetic resonance (NMR) experiments at elevated temperatures (40, 55, and 70 °C, Figure S1). Thus, we hypothesized the formation of rotational isomers due to the increased steric hindrance of 2-picoline in PicoCarbo(IV) derivatives compared to PicoCarbo(II).

### Interaction of Platinum(II) Complexes with GSH

**Figure 1:**
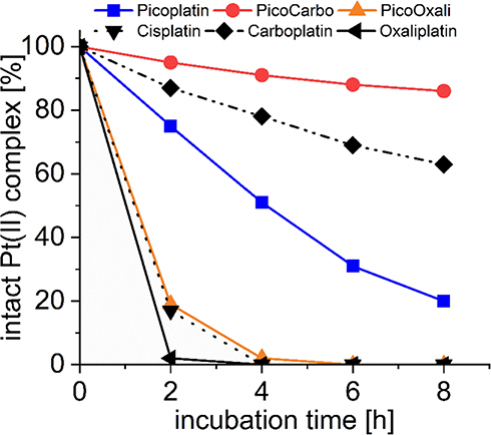
Interaction of 1 mM picoplatin, cisplatin, **PicoCarbo,** and carboplatin in 10% D_2_O/90% PBS (pH 7.4) or 1 mM **PicoOxali** and oxaliplatin in 10% D_2_O/90% PB (pH 7.4) with 10 equiv GSH at 37 °C over 8 h, respectively. Reactions were monitored with ^1^H NMR or [^1^H, ^15^N] HSQC NMR for cisplatin.

Picoplatin was originally designed as a sterically hindered cisplatin, where the bulky 2-picoline ligand should protect the platinum(II) core from rapid deactivation by elevated levels of sulfur-containing biomolecules like GSH.^13^ Accordingly, the reaction between GSH and picoplatin in a 1:1 molar ratio has been studied.^46^ Surprisingly, to the best of our knowledge, a direct comparison with the clinically used platinum drugs was not reported in literature so far. Thus, we investigated the incubation of picoplatin, **PicoCarbo**, and **PicoOxali** in comparison to ^15^N-cisplatin, carboplatin, and oxaliplatin with a 10-fold excess of GSH in a mixture of 10% D_2_O/90% phosphate buffer (PB) or phosphate-buffered saline (PBS) at pH 7.4 and 37 °C over 8 h. The amount of remaining platinum(II) complex was monitored via ^1^H NMR spectroscopy and, particularly for ^15^N-cisplatin, via [^1^H, ^15^N] heteronuclear single quantum coherence (HSQC) NMR spectroscopy (Figure 1).^47^

According to literature, cisplatin,^48^ carboplatin,^49^ and picoplatin^50^ are widely stable in commercial 0.9% NaCl solutions, but it is known for oxaliplatin that the oxalate is readily replaced by chlorido ligands.^51^ Thus, in order to more accurately study the different reaction kinetics of the platinum(II) complexes with GSH, first their stability was investigated in either PBS or PB (Figures S2 and S3). Indeed, while picoplatin, ^15^N-cisplatin, carboplatin, and **PicoCarbo** were widely stable in 10% D_2_O/90% PBS over 8 h incubation at 37 °C (>95% left, Figure S2), oxaliplatin and **PicoOxali** were only stable in 10% D_2_O/90% PB (>90% left) and readily degraded in 10% D_2_O/90% PBS (Figure S3). Subsequently, all platinum(II) complexes were coincubated with 10 equiv of GSH in their most stable buffered solutions (Figure 1). Picoplatin decayed at a moderate rate, with ∼50% reacted after 4 h and ∼20% intact platinum(II) complex after 8 h incubation (Figure S4). In comparison, ^15^N-cisplatin reacted much faster in the presence of GSH with only ∼20% left after 2 h and a nearly complete reaction after 4 h, which is in agreement to literature (Figure S5).^52^ Thus, the introduction of the 2-picoline ligand and the resulting increase in steric hindrance indeed strongly reduced the reaction rate with GSH. **PicoCarbo** reacted very slowly with GSH, with still ∼90% left after 8 h. Again, the approved platinum(II) reference drug (carboplatin) decayed considerably faster, with ∼60% intact complex left after 8 h of incubation. However, it has to be intriguingly noted that carboplatin was much more resistant to interaction with GSH than picoplatin, which was specifically developed to prevent interaction with GSH. The higher stability of the 2-picoline-containing complex was similarly observed when comparing **PicoOxali** to oxaliplatin. Interestingly, however, both were among the least stable complexes overall.

### Hydrolytic Stability and Reduction Kinetics of Platinum(IV)-Succinimide Complexes

**Figure 2:**
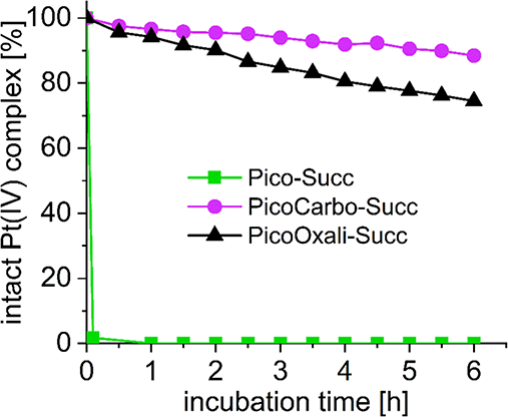
Reduction kinetics of 1 mM **Pico-Succ**, **PicoCarbo-Succ**, and **PicoOxali-Succ** in 150 mM PB (pH = 7.4) at 20 °C with 10 equiv of AA over 6 h, measured with UHPLC.

Platinum(IV) prodrugs need to be reduced in the tumor environment to release the cytotoxic platinum(II) complexes. Thus, the reduction properties of **Pico-Succ**, **PicoCarbo-Succ,** and **PicoOxali-Succ** were investigated. As mentioned above, succinimide complexes were used for these studies to avoid competitive hydrolysis of the maleimide moiety. All platinum(IV)-succinimide complexes were incubated in both the absence and presence of a 10-fold excess l-ascorbic acid (AA) in PB at pH 7.4 and 20 °C over 6 h. The amount of remaining intact platinum(IV) complex at given time points was measured via ultrahigh performance liquid chromatography (UHPLC), as shown in Figure 2. **Pico-Succ** was reduced extremely fast, with only traces of the original complex left in the first measurement after ∼5 min of incubation. Even with equimolar amounts of AA, 90% of **Pico-Succ** was already reduced after ∼5 min (Figure S6). This level of reductive instability is exceptionally high, even when compared to the “fast” reducing cisplatin(IV) prodrugs, which show a complete conversion after ∼10 h in the presence of 10 equiv AA.^41,53^ Notably, **Pico-Succ** was stable in the absence of AA under the same conditions for >6 h (Figure S7).

It is well-known that platinum(IV) complexes derived from, e.g., oxaliplatin or carboplatin, bearing leaving groups other than chlorido ligands, have distinctly slower reduction rates compared to cisplatin(IV) analogs.^54^ This can be explained by a bridging interaction between the chlorido ligands and the reducing agent, which facilitates electron transfer to the platinum center.^55^ Indeed, **PicoOxali-Succ** and **PicoCarbo-Succ** were more stable, with ∼70% and even ∼90% left after 6 h of incubation, respectively (Figure 2). The reductive behavior was also investigated in the presence of 10 equiv GSH in coherence with the above investigated interactions of platinum(II) with GSH (Figure S8). It is generally accepted that platinum(IV) complexes are faster reduced with AA in comparison with GSH, and indeed, no notable reduction was observed for **PicoCarbo-Succ** and **PicoOxali-Succ** over 6 h of incubation. In contrast, **Pico-Succ** was again nearly instantly reduced with only traces of the original compound remaining after 5 min, confirming the extremely low reductive stability.

### In Vitro Anticancer Activity of Platinum(II) and Platinum(IV)-Succinimide Complexes

**Figure 3:**
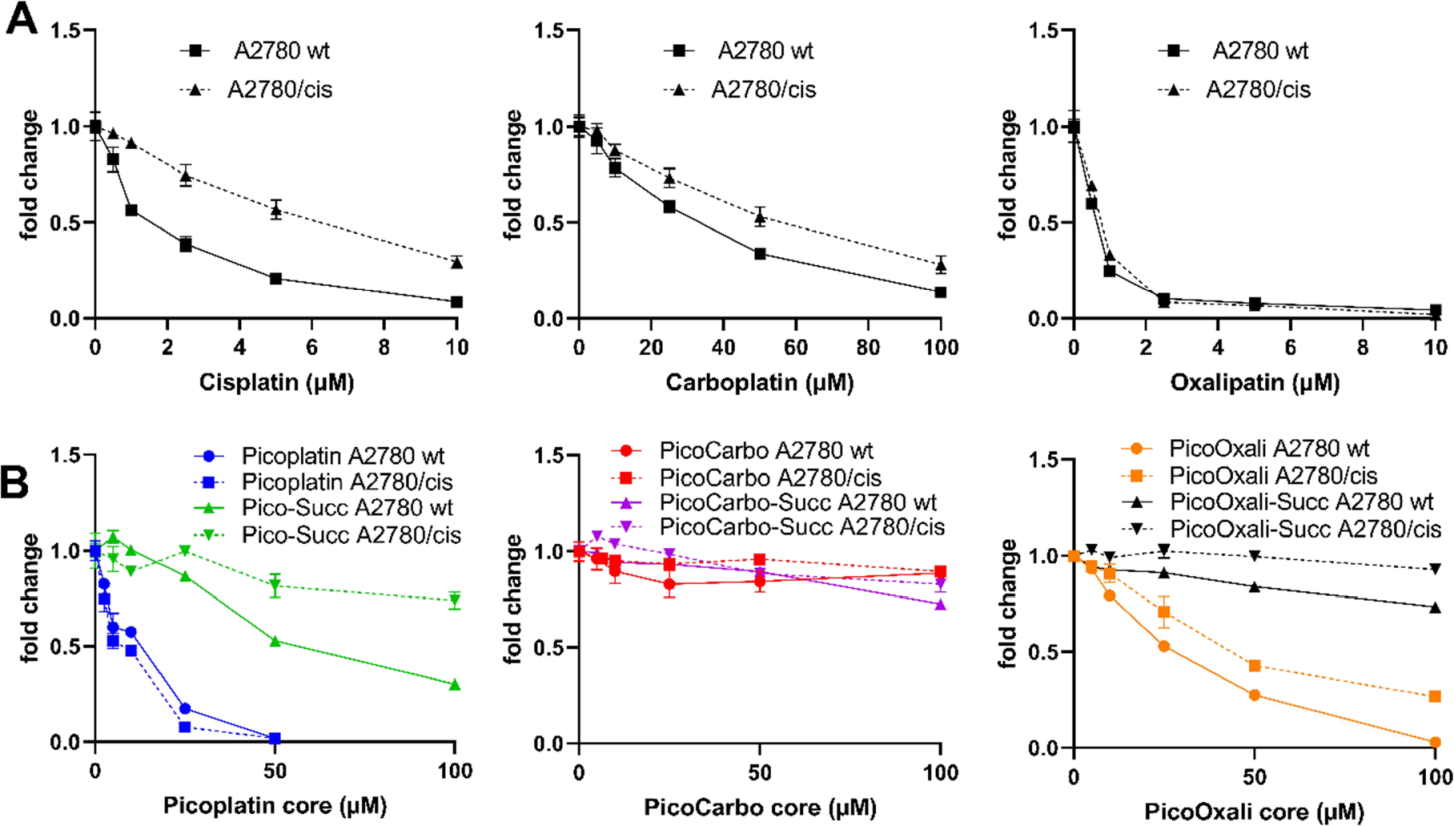
Anticancer activity of (A) approved platinum(II) drugs and (B) picoplatin(II) derivatives and their corresponding platinum(IV)-succinimides against A2780/wt and A2780/cis cancer cells after 72 h was determined via MTT-based assays. Values refer to means ± SD of one representative experiment performed in triplicates.

To evaluate the anticancer activity of the platinum complexes and to assess the impact of varying intracellular GSH levels, the human ovarian cancer cell line A2780/wt and its cisplatin-resistant subline A2780/cis, which is characterized (among other changes) by elevated intracellular GSH levels,^56^ were used for a first screening. Cells were treated with the new platinum(II) and platinum(IV)-succinimide complexes as well as picoplatin, cisplatin, carboplatin, and oxaliplatin, and their viability was measured after 72 h using an MTT-based assay (Figure 3 and Table 1). As expected, cisplatin displayed significantly higher IC_50_ values (5.1-fold) in the A2780/cis cells compared to the A2780/wt cells, confirming their resistance. Moreover, A2780/cis cells were cross-resistant to carboplatin (2.3-fold). In contrast, oxaliplatin treatment resulted in similar IC_50_ values (1.1-fold), while the A2780/cis model had a nonsignificant tendency toward collateral sensitivity against picoplatin (0.7-fold). These effects are in good agreement with reports in the literature.^15,56^ With regards to our new platinum(II) drugs, rather unexpectedly, both picoplatin hybrid drugs were distinctly less active than picoplatin (3.5-fold and >10-fold, respectively). Moreover, **PicoOxali** activity was slightly but not significantly reduced in A2780/cis (1.4-fold), indicating some degree of cross-resistance (in contrast to picoplatin and oxaliplatin). **PicoCarbo** showed no effect on cell viability in both cell lines (up to 100 μM). Consequently, no conclusion on the impact of the cisplatin-mediated resistance mechanisms could be drawn for this compound.

**Table 1:** IC_50_ Values for the Tested Platinum Complexes in A2780/wt and A2780/cis Ovarian Cancer Cells after 72 h of Drug Exposure^a^^,^^b^^,^^c^

	A2780/wt (72 h)			A2780/cis (72 h)			
	IC_50_ (μM)	SD	*n*	IC_50_ (μM)	SD	*n*	RF
cisplatin	**1.54**	0.16	4	**7.81**	0.68	5	5.1****
oxaliplatin	**0.63**	0.14	3	**0.68**	0.02	4	1.1^ns^
carboplatin	**32.05**	1.68	4	**74.91**	3.96	4	2.3****
picoplatin	**9.48**	1.33	3	**6.19**	0.92	3	0.7^ns^
PicoCarbo	**>100**		3	**>100**		3	
PicoOxali	**33.42**	6.17	3	**45.97**	4.11	3	1.4^ns^
Pico-Succ	**63.18**	11.90	3	**>100**		4	>1.6
PicoCarbo-Succ	**>100**		3	**>100**		3	
PicoOxali-Succ	**>100**		3	**>100**		3	

a All values given are means ± SD of independent experiments.

b Resistance factor (RF) for A2780/wt cells was calculated with the fold-change between IC_50_ values of A2780/cis:A2780/wt.

c Significance of IC_50_ values of a drug between A2780/wt vs A2780/cis was calculated by unpaired two-sided t-test with Welch̀s correction (****p < 0.0001 and ns = not significant).

The platinum(IV)-succinimide complexes were generally less active than the respective platinum(II) drugs, which is in good agreement with their expected prodrug nature.^42,57^ In detail, **Pico-Succ** was the only complex for which IC_50_ values could be calculated. In contrast, **PicoCarbo-Succ** and **PicoOxali-Succ** displayed no cytotoxicity up to concentrations of 100 μM. These data are in good agreement with the distinctly faster reduction of **Pico-Succ** compared to the other derivatives (Figure 2).

Due to the clinical investigation of picoplatin against colorectal cancer,^21,22^ the in vitro antitumor activity was also assessed against two colon cancer models: HCT116 (human) and CT26 (murine) (Table 2A). In the HCT116 model, only picoplatin exhibited activity (IC_50_ of 22.5 μM), while the other platinum(II) derivatives showed no impact on cell viability up to 100 μM. In the CT26 model, the activity profile was comparable to that observed in the A2780 ovarian cancer cells, with the exception of **PicoCarbo** exhibiting measurable anticancer activity (IC_50_ 74.5 μM). To investigate if prolonged incubation allows (pro)drug activation, long-term colony formation assays with the two colon cancer models were conducted with measurement of the cell clone number after 7 days (Table 2B). Interestingly, extended treatment time resulted in enhanced activity for all platinum(II) drugs (especially for cisplatin, carboplatin, and picoplatin), while of the platinum(IV) drugs, only **Pico-Succ** exhibited higher activity (reduction of IC_50_ values by >2–3-fold). In contrast, **PicoCarbo-Succ** and **PicoOxali-Succ** again showed no activity up to 100 μM. This indicates that their lower reduction rate probably prevents their activation under cell culture conditions.

**Table 2:** IC_50_ Values for the Tested Platinum Complexes in HCT116 and CT26 Colon Cancer Cells after (A) 72 h and (B) 7 Days Drug Exposure, Measured by MTT and Clonogenic Assay, Respectively^a^

A	HCT116 (72 h)			CT26 (72 h)		
	IC_50_ (μM)	SD	*n*	IC_50_ (μM)	SD	*n*
cisplatin	**3.15**	1.68	4	**2.17**	0.36	3
oxaliplatin	**2.19**	0.18	3	**1.97**	0.25	3
carboplatin	**63.21**	13.71	4	**22.10**	1.70	3
picoplatin	**22.50**	2.18	3	**11.94**	0.33	3
PicoCarbo	**>100**		3	**74.49**	4.62	3
PicoOxali	**>100**		3	**33.70**	3.71	3
Pico-Succ	**>100**		3	**77.84**	15.61	3
PicoCarbo-Succ	**>100**		3	**>100**		3
PicoOxali-Succ	**>100**		3	**>100**		3

B	HCT116 (7 days)			CT26 (7 days)		
	IC_50_ (μM)	SD	*n*	IC_50_ (μM)	SD	*n*
cisplatin	**0.82**	0.04	3	**0.46**	0.02	3
oxaliplatin	**1.46**	0.20	3	**1.81**	0.44	3
carboplatin	**7.41**	0.80	3	**4.37**	0.27	3
picoplatin	**3.39**	0.42	3	**2.90**	0.21	3
PicoCarbo	**89.95**	4.98	3	**26.30**	6.40	3
PicoOxali	**15.82**	5.97	3	**7.26**	0.36	3
Pico-Succ	**32.06**	3.35	3	**30.90**	5.61	3
PicoCarbo-Succ	**>100**		1	**>100**		3
PicoOxali-Succ	**>100**		1	**>100**		2

a All values given are means ± SD of independent experiments.

### Albumin-Binding Kinetics of Platinum(IV)-Maleimide Complexes

**Figure 4:**
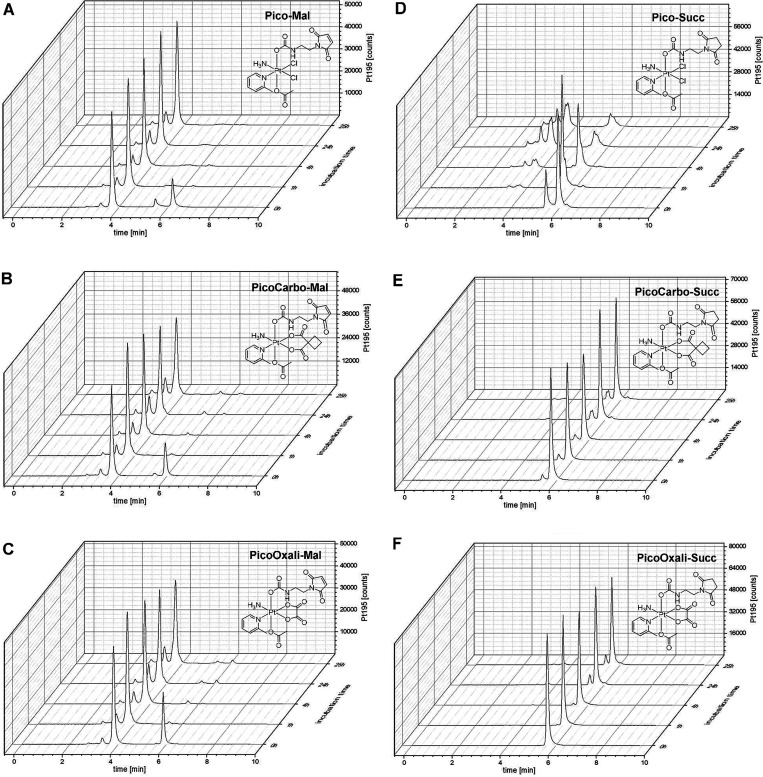
^195^Pt traces of incubation of 50 μM (A) **Pico-Mal**, (B) **PicoCarbo-Mal**, (C), **PicoOxali-Mal**, (D) **Pico-Succ**, (E) **PicoCarbo-Succ**, and (F) **PicoOxali-Succ** in FCS (containing 150 mM PB, pH = 7.4) at 37 °C over 24 h, measured with SEC-ICP-MS.

The albumin-binding properties of the platinum(IV)-maleimide derivatives **Pico-Mal**, **PicoCarbo-Mal**, and **PicoOxali-Mal** were studied in comparison to the platinum(IV)-succinimide complexes (unable to bind to thiol groups) by incubation in phosphate-buffered fetal calf serum (FCS) at 37 °C over 25 h. ^195^Pt traces were measured via size-exclusion chromatography coupled to inductively coupled plasma mass spectrometry (SEC-ICP-MS; Figure 4A–F). Serum proteins have a retention range of 2–4 min, with albumin eluting at 4.0 min (Figure S9) as part of the high-molecular-weight fraction (HMWF), while the low-molecular-weight fraction (LMWF) elutes after 5 min. Already at the 0 h time point, approximately 70% of all three platinum(IV)-maleimide prodrugs were bound to albumin, which increased to >97% after 1 h incubation. The remaining 2–3% are most likely traces of hydrolyzed maleimide complexes unable to bind to albumin. For the reference succinimide compounds **PicoCarbo-Succ** and **PicoOxali-Succ**, no albumin conjugation could be observed as expected and ^195^Pt could only be detected in the LMWF (Figure 4E,F). Noteworthy, **Pico-Succ** was highly unstable, with only traces of intact platinum(IV) complex left after 1 h incubation (Figure 4D). Interestingly, the overall pattern looked quite similar to the SEC-ICP-MS measurements of picoplatin in FCS (Figure S10), with the formation of multiple protein adducts over time. Although a different intermediate at ∼5.6 min was formed in the case of **Pico-Succ**, this similarity can most probably be explained by the fast reduction of **Pico-Succ**. Reduction solely of **Pico-Succ** in serum but not **PicoCarbo-Succ** and **PicoOxali-Succ** is very well in agreement with the studied reduction kinetics in the presence of AA (Figure 2) and GSH (Figure S8). Finally, these data clearly highlight that the albumin conjugation of **Pico-Mal** leads to a very stable adduct unaffected by (fast) reduction processes, whereas free **Pico-Succ** is not stable in serum and is readily reduced to picoplatin.

### Serum Pharmacokinetics, Organ Distribution, and In Vivo Anticancer Activity

Following the albumin-binding studies, the pharmacological behavior, organ distribution, and anticancer efficacy of the picoplatin(IV)-maleimide complexes were studied in comparison to their platinum(II) counterparts in Balb/c mice bearing CT26 tumors. The CT26 model was chosen due to its moderate-to-strong in vitro response to the picoplatin(II) derivatives (Table 1) and its high propensity for albumin accumulation in vivo.^42^ Additionally, CT26-bearing mice are a syngeneic model with an intact immune system, which is an essential aspect for evaluating the full therapeutic potential of platinum-based drugs.^58^ Of note, CT26 allografts are only moderately responsive to oxaliplatin and highly carboplatin-resistant (Figure S11).

**Figure 5:**
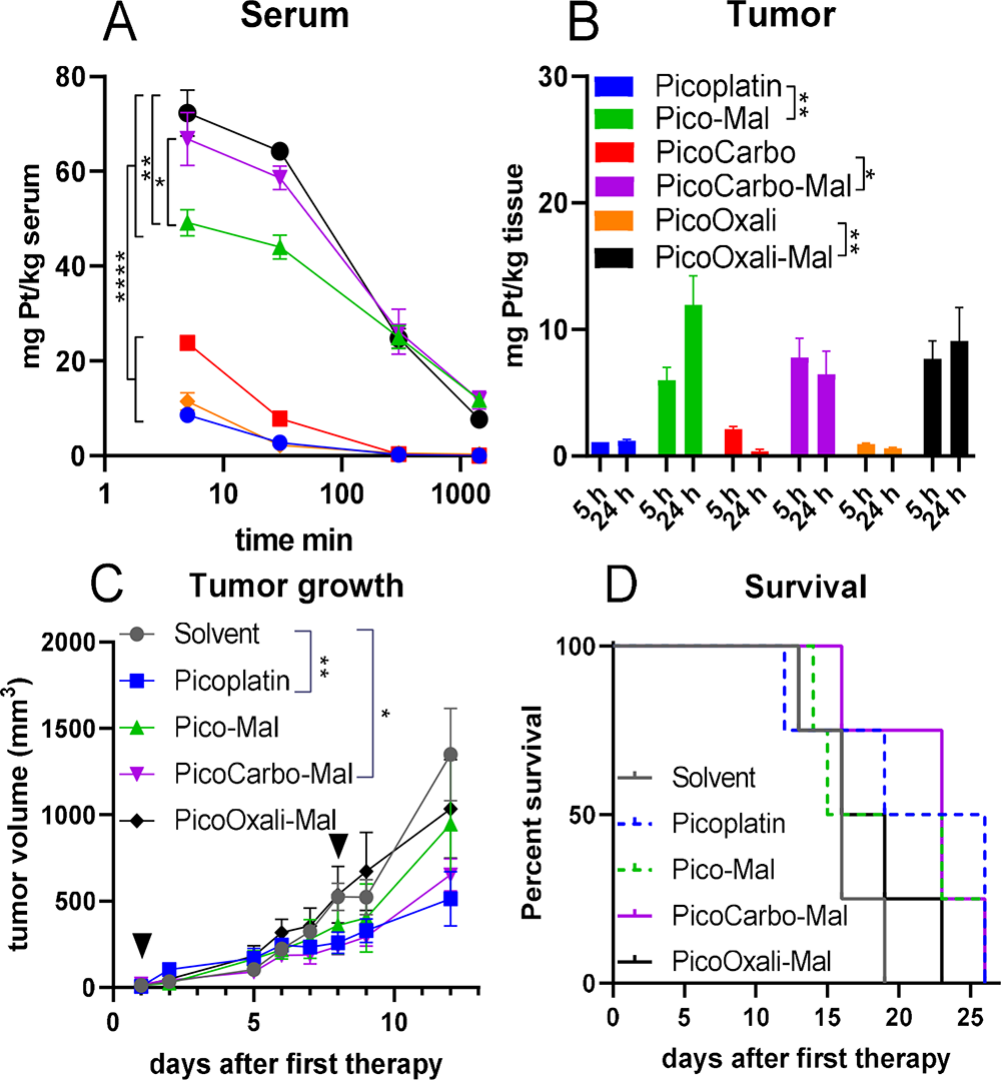
Pharmacological evaluation and anticancer activity of the platinum complexes in CT26-bearing Balb/c mice. For the pharmacological studies, animals were treated once i.v. with concentrations equimolar to 20 mg/kg picoplatin. (A) Serum samples were collected after 5, 30, 300, and 1440 min; (B) tumor samples were collected after 5 and 24 h. Platinum levels of all samples were measured via ICP-MS. For evaluation of anticancer activity, CT26-bearing Balb/c mice were treated once a week for 2 weeks (marked with an arrow) i.v. with concentrations equimolar to 20 mg/kg picoplatin. (C) Impact on tumor growth; data shown until first animal had to be sacrificed. (D) Overall survival is depicted via a Kaplan–Meier curve. Data given in A–C are means ± SEM. Statistical significance was tested by two-way ANOVA (*p < 0.05, **p < 0.01, ***p < 0.001, ****p < 0.0001).

The pharmacological experiments indicated that all three maleimide compounds had an improved pharmacokinetic profile in agreement with previous studies on maleimide-containing, oxaliplatin-releasing platinum(IV) derivatives.^42,59^ In detail, the maleimide functionalization led to significantly prolonged and elevated drug levels in serum (Figure 5A) and also distinctly increased drug levels in tumor tissues (∼6-fold and ∼14-fold after 5 and 24 h, respectively) compared to the corresponding platinum(II) complexes (Figures 5B and S12). This behavior aligns with the binding of the maleimide-functionalized platinum(IV) complexes to endogenous albumin,^36^ which facilitates drug accumulation in the malignant tissue via the EPR effect.^37^ Additionally, this accumulation is supported by the enhanced nutrient demands of the tumor.^38^ Interestingly, in the case of **Pico-Mal**, the drug serum levels at the 5 min time point were lower compared to PicoOxali and PicoCarbo. The concurrently observed higher platinum levels in the kidneys suggest a slower in vivo albumin-binding process and a faster excretion rate of **Pico-Mal**.

For albumin-binding drugs, in vitro IC_50_ values often fail to predict in vivo anticancer activity accurately. This discrepancy arises because these drugs build a depot in the malignant tissue, enabling sustained prodrug activation over time, in contrast to untargeted small molecules, which typically lack such tumor-specific accumulation. Consequently, we decided to test all maleimide-functionalized compounds in vivo, despite the rather weak anticancer activity of **PicoCarbo-Succ** and **PicoOxali-Succ** in cell culture. Of note, only **PicoCarbo-Mal** and picoplatin(II) significantly inhibited tumor growth, which however did not translate into enhanced long-term anticancer effects (Figure 5C,D). The data on **Pico-Mal** are especially noteworthy, as the succinimide derivative was active in the cell culture against the same cancer cell model. However, despite the good pharmacological profile, **Pico-Mal** was disappointingly inactive (in contrast to picoplatin) in the in vivo activity experiment. To the best of our knowledge, this represents the first example of a maleimide-coupled platinum(IV) complex, where the respective platinum(II) complex has superior anticancer properties.

### Prodrug Activation By Cytochrome C

**Figure 6:**
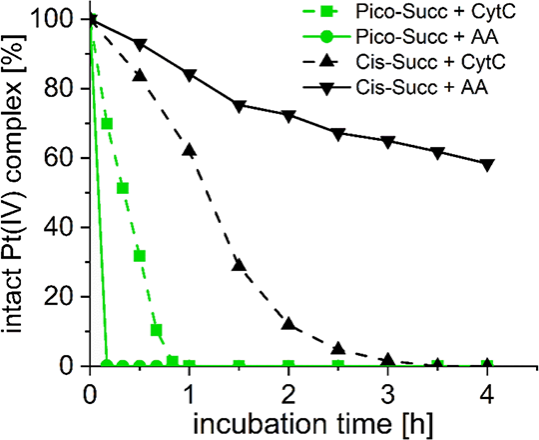
Reduction kinetics of 700 μM **Pico-Succ** and **Cis-Succ** in 50 mM PB (pH = 6.0) at 37 °C with 0.1 equiv CytC and 10 equiv NADH in comparison to 10 equiv AA, measured with UHPLC.

To explain the rather unusual in vivo findings, especially with respect to **Pico-Mal**, we hypothesized that inefficient reductive release of the platinum(II) core from albumin due to steric hindrance of the 2-picoline could provide a plausible explanation for the lack of antitumor activity despite a favorable pharmacokinetic profile. Gibson and coworkers already showed that metalloproteins (e.g., hemoglobin or cytochrome C) of the HMWF in cell extracts are in fact far more efficient in the reduction of platinum(IV) prodrugs compared to the LMWF containing small molecule reductants such as GSH and AA.^55^ To gain insights into whether the molecular weight and thus steric demands of the reducing agent impact the reduction of picoplatin(IV) derivatives, **Pico-Succ** and the reference complex **Cis-Succ** were analyzed in the presence of cytochrome C (CytC; ∼12,000 g/mol) or AA (176 g/mol). Therefore, **Pico-Succ** and **Cis-Succ** (for synthesis and ^1^H NMR characterization, see experimental part and Scheme S1) were coincubated at pH 6.0 with 0.1 equiv CytC^60^ and 10 equiv reduced NADH or with 10 equiv AA at 37 °C over 4 h, and the reduction kinetics were compared relatively. The amount of remaining intact platinum(IV) complex at given time points was measured via UHPLC (Figure 6). Indeed, in agreement with literature,^61^**Cis-Succ** was reduced distinctly faster in the presence of CytC with complete reduction after 4 h, whereas incubation with AA only led to ∼40% reduction at that time point. In contrast, **Pico-Succ** reduced much slower in the presence of CytC than AA, with complete reduction only after 1 h incubation compared to immediate reduction with AA. Although in absolute values, **Pico-Succ** was still faster reduced than **Cis-Succ**, these results confirm that the 2-picoline moiety of picoplatin(IV) significantly impacts the reduction rate with the model high-molecular weight reductant CytC compared to cisplatin(IV), presumably due to sterically hindered electron transfer. This effect could be even more pronounced upon albumin binding and possibly serve as one parameter for the lack of in vivo anticancer activity. However, further investigations are needed to better understand these observations.

## Conclusions

Platinum(II) complexes are essential treatments against a broad variety of cancers but come with severe adverse effects and tumor resistance. Picoplatin was developed as a bulky cisplatin derivative to suppress deactivation by increased levels of GSH and overcome drug resistance.^9,62^ Although picoplatin has been investigated in clinical trials, it was not approved due to the lack of an increased overall survival. Platinum(IV) complexes based on picoplatin are strongly underinvestigated. Hence, we designed a series of albumin-targeted platinum(IV) prodrugs based on picoplatin and two “hybrid” derivatives **PicoOxali** and **PicoCarbo**. Although picoplatin(IV) was rapidly reduced compared to PicoOxali(IV) and PicoCarbo(IV), all maleimide-bearing complexes formed stable albumin adducts. This also translated into promising in vivo pharmacokinetic profiles, with strongly enhanced plasma levels and tumor accumulation in comparison to their respective platinum(II) counterparts. However, the in vivo anticancer activity did not meet our expectations. We recently published several carboplatin(IV)- and oxaliplatin(IV)-maleimide complexes with similar pharmacokinetic properties in vivo, resulting in distinctly improved tumor growth inhibition and long-term survival.^41,53,59^ This is the first example in which promising results in pharmacological studies did not corroborate with the anticancer activity in vivo. The underlying reasons seem to be multifaceted. Interaction studies confirmed that the bulky 2-picoline moiety in picoplatin(II) distinctly decelerates the reaction/deactivation with GSH compared to cisplatin. This very beneficial effect for overcoming GSH-related platinum(II) drug resistance is presumably a drawback for activating picoplatin(IV) compounds. Reduction data using CytC as a model system instead of AA suggest that such high-molecular weight reducing agents (primarily responsible for platinum(IV) reduction in vivo) activate picoplatin(IV) much less efficiently than cisplatin(IV) complexes. An even stronger effect of the 2-picoline moiety on the activation by reduction process can be expected in albumin-bound picoplatin(IV) complexes, with higher steric hindrance due to the protein surface. Furthermore, for **Pico-Mal** lower initial serum levels and high kidney accumulation could be observed. This indicates differences in the pharmacology between the platinum(IV)-maleimide complexes, which could be related to the low in vivo activity. Finally, the reactivity of the released platinum(II) complex may also contribute to the anticancer efficiency. Taken together, our data demonstrate that the design of platinum(IV)-based prodrugs for drug delivery systems requires careful consideration of multiple parameters. This is not only the case for albumin-targeted complexes but also for other nanocarrier-based strategies.

## Experimental Section

### Synthesis: Materials and Methods

Potassium tetrachloridoplatinate was purchased from Johnson Matthey (Switzerland). Water for the synthesis was taken from a reverse osmosis system. Reactions were conducted under atmospheric conditions and at room temperature (RT) unless stated otherwise. For HPLC measurements, Milli-Q water (18.2 MΩ·cm, Merck Milli-Q Advantage, Darmstadt, Germany) was used. Chemicals and solvents were purchased from commercial suppliers (Sigma-Aldrich, Merck, Acros, Fluka, and FisherScientific). *Cis*-[PtI_2_(NH_3_)(2-Pic)],^30^ picoplatin,^30^ 1-(2-isocyanatoethyl)-1*H*-pyrrole-2,5-dione,^39^ 1-(2-isocyanatoethyl)pyrrolidine-2,5-dione,^39^ and *cis*-[PtCl_2_(^15^NH_3_)_2_]^46^ were synthesized, as described in the literature. Electrospray ionization (ESI) mass spectra were recorded on a Bruker Amazon SL ion trap mass spectrometer in positive and negative mode by direct infusion at the Mass Spectrometry Centre of the University of Vienna. One- and two-dimensional ^1^H, ^15^N, and ^13^C spectra were recorded on a Bruker AV Neo 500 or AV III 600 spectrometer at 298 K. For ^1^H and ^13^C NMR spectra, the solvent residual peak was taken as an internal reference. The ^1^H and ^13^C NMR spectra of the final compounds are depicted in Figures S13–S18. Purification by preparative reverse phase (RP) HPLC was performed on an Agilent 1200 series system using a Waters XBridge C18 column (19 × 250 mm). Elemental analysis measurements were carried out on a PerkinElmer 2400 CHN elemental analyzer at the Microanalytical Laboratory of the University of Vienna and are within ±0.4%, confirming >95% purity. The content of TFA and water can vary between different batches of the same compound.

### General Procedure A: Synthesis of Platinum(II) Hybrid Complexes

*Cis*-[PtI_2_(NH_3_)(2-Pic)] was suspended in MQ-H_2_O (0.5 M), silver cyclobutyl dicarboxylate^43^ or silver oxalate^43^ (1 equiv) was added, and the resulting mixture was left stirring overnight at RT in the dark. The solvent was removed under reduced pressure, and the crude product was purified via preparative RP-HPLC.

### General Procedure B: Asymmetric Oxidation of Platinum(II) Complexes

The respective platinum(II) complex was dissolved in acetic acid (0.15 M), hydrogen peroxide was added (50 wt % solution in MQ-H_2_O, 10 equiv), and the resulting mixture was stirred for 1.5 h in the dark at RT. The solvent was removed under reduced pressure, the crude was suspended in ethanol, and dried again under reduced pressure (repeated twice). The crude product was purified via silica flash column chromatography or preparative RP-HPLC.

### General Procedure C: Synthesis of Platinum(IV)-Maleimide or Platinum(IV)-Succinimide Complexes

**Pt(IV)-OAc-OH** was dissolved in anhydrous DMF (0.5 M) under an argon atmosphere at RT. 1-(2-Isocyanatoethyl)-1*H*-pyrrole-2,5-dione or 1-(2-isocyanatoethyl)pyrrolidine-2,5-dione (1.5 equiv) was added, and the resulting mixture was stirred at RT in the dark for 17 h (overnight). DMF was removed under high vacuum at 40 °C, and the crude product was purified via preparative RP-HPLC.

#### (*SP*-4-4)[(Ammine)(cyclobutane-1,1-dicarboxylato)(2-methylpyridine)platinum(II)] (PicoCarbo)

Synthesized according to general procedure **A**. The crude product was purified via preparative RP-HPLC (6% MeCN (+0.1% HCOOH) in H_2_O (+0.1% HCOOH); isocratic) and lyophilized to obtain the title compound as a white solid (258.6 mg, 34%). ^1^H NMR (500 MHz, DMSO-*d*_6_) δ 8.87–8.79 (d, *J* = 6.0 Hz, 1H, C*H*_Pic_), 7.90–7.84 (t, *J* = 7.7 Hz, 1H, C*H*_Pic_), 7.56–7.50 (d, *J* = 7.8 Hz, 1H, C*H*_Pic_), 7.37–7.30 (t, *J* = 6.7 Hz, 1H, C*H*_Pic_), 4.56–4.24 (bs, 3H, N*H*_3_), 3.01 (s, 3H, C*H*_3,Pic_), 2.81–2.72 (m, 2H, C*H*_2,Carbo_), 2.71–2.61 (m, 2H, C*H*_2,Carbo_), 1.75–1.62 (m, 2H, C*H*_2,Carbo_) ppm. MS (*m*/*z*): calcd C_12_H_16_N_2_O_4_NaPt (M + H)^+^, 470.07; found, 470.10. EA calcd C_12_H_16_N_2_O_4_Pt·1H_2_O: C, 30.97; H, 3.90; N, 6.02; found: C, 30.80; H, 3.71; N, 6.05.

#### (*SP*-4-4)(Ammine)(2-methylpyridine)(oxalato)platinum(IV)] (PicoOxali)

Synthesized according to general procedure **A**. The crude product was purified via preparative RP-HPLC (2% MeOH (+0.1% HCOOH) in H_2_O (+0.1% HCOOH); isocratic) and lyophilized to obtain the title compound as a white solid (536 mg, 43%). ^1^H NMR (500 MHz, DMSO-*d*_6_) δ 8.92–8.80 (d, *J* = 5.8 hz, 1H, C*H*_Pic_), 7.93–7.85 (t, *J* = 7.7 Hz, 1H, C*H*_Pic_), 7.60–7.51 (d, *J* = 7.6 Hz, 1H, C*H*_Pic_), 7.39–7.31 (t, *J* = 6.6 Hz, 1H, C*H*_Pic_), 4.83–4.40 (bs, 3H, N*H*_3_), 3.02 (s, 3H, C*H*_3,Pic_) ppm. MS (*m*/*z*): calcd C_8_H_10_N_2_O_4_NaPt (M + H)^+^, 416.02; found, 416.06. EA calcd C_8_H_10_N_2_O_4_Pt·0.5TFA·0.5H_2_O: C, 23.54; H, 2.52; N, 6.10; found: C, 23.40; H, 2.49; N, 6.40.

#### (OC-6-21)[Acetato(ammine)(dichlorido)(hydroxido)(2-methylpyridine)platinum(IV)] (Pico-OH-OAc)

Synthesized according to general procedure **B**. The crude product was purified via silica flash column chromatography (MeOH:EtOAc 40:60) to obtain the title compound as a green-white solid (34.5 mg, 79%). ^1^H NMR (500 MHz, DMSO-*d*_6_) δ 8.98–8.74 (m, 1H, C*H*_Pic_), 8.08–7.96 (t, *J* = 7.6 Hz, 1H, C*H*_Pic_), 7.58–7.45 (m, 2H, C*H*_Pic_), 6.90–6.43 (m, 3H, N*H*_3_), 3.00–2.66 (m, 3H, C*H*_3,Pic_), 1.91 (s, 3H, OCOC*H*_3_) ppm. MS (*m*/*z*): calcd C_8_H_14_Cl_2_N_2_O_3_NaPt (M + Na)^+^, 474.99; found, 475.02.

#### (OC-6-21)[Acetato(ammine)(cyclobutane-1,1-dicarboxylato)hydroxidoplatinum(IV)] (PicoCarbo-OH-OAc)

Synthesized according to general procedure **B**. The crude product was purified via preparative RP-HPLC (9% MeCN (containing 0.1% CF_3_COOH) in H_2_O (containing 0.1% CF_3_COOH); isocratic) and lyophilized to obtain the title compound as a white solid (62.0 mg, 38%). ^1^H NMR (500 MHz, DMSO-*d*_6_, major isomer A, minor isomer B) δ 8.57–8.43 (m, 2H, C*H*_Pic_, A + B), 8.14–8.03 (m, 2H, C*H*_Pic_, A + B), 7.64–7.41 (m, 4H, C*H*_Pic_, A + B), 6.64–6.17 (m, 6H, N*H*_3_, A + B), 2.82–2.74 (m, 6H, C*H*_3,Pic_, A + B), 2.60–2.41 (m, 6H, C*H*_2,Carbo_, A + B), 1.99 (s, 3H, OCOC*H*_3_, A), 1.89 (s, 3H, OCOC*H*_3_, B), 1.90–1.63 (m, 6H, C*H*_2,Carbo,_ A + B) ppm. MS (*m*/*z*): calcd C_14_H_20_N_2_O_7_NaPt (M + Na)^+^, 546.08; found, 546.12.

#### (OC-6-21)[Acetato(ammine)hydroxido(oxalato)platinum(IV)] (PicoOxali-OH-OAc)

Synthesized according to general procedure **B**. The crude product was purified via preparative RP-HPLC (5% MeCN (containing 0.1% HCOOH) in H_2_O (containing 0.1% HCOOH); isocratic) and lyophilized to obtain the title compound as a white solid (408.0 mg, 64%). ^1^H NMR (500 MHz, DMSO-*d*_6_) δ 8.56–8.47 (d, *J* = 6.4 Hz, 1H, C*H*_Pic_), 8.14–8.06 (t, *J* = 7.7 Hz, 1H, C*H*_Pic_), 7.61–7.52 (m, 2H, C*H*_Pic_), 6.70–6.30 (m, 3H, N*H*_3_), 2.81 (s, 3H, C*H*_3,Pic_), 1.89 (s, 3H, OCOC*H*_3_) ppm. MS (*m*/*z*): calcd C_10_H_14_N_2_O_7_NaPt (M + H)^+^, 492.03; found, 492.06.

#### (OC-6-21)[Acetato(ammine)(dichlorido)(1-(2-isocyanatoethyl)pyrrolidine-2,5-dione)(2-methylpyridine)platinum(IV)] (Pico-Succ)

Synthesized according to general procedure **C**. The crude product was purified via preparative RP-HPLC (22% MeCN (containing 0.1% CF_3_COOH) in H_2_O (containing 0.1% CF_3_COOH); isocratic) and lyophilized to obtain the title compound as a yellow-white solid (37.0 mg, 69%). ^1^H NMR (500 MHz, DMSO-*d*_*6*_) δ 8.84–8.55 (m, 1H, C*H*_Pic_), 8.14–8.03 (t, *J* = 7.5 Hz, 1H, C*H*_Pic_), 7.73–7.39 (m, 5H, C*H*_Pic_ + N*H*_3_), 6.86–6.78 + 6.31–6.21 (m, 1H, N*H*_Ca_), 3.49–3.40 (m, 1H, C*H*N(CO)_2_), 3.39–3.23 (m, 1H, C*H*N(CO)_2_), 3.21–3.11 (m, 1H, C*H*NH_Ca_), 3.02–2.92 (m, 1H, C*H*NH_Ca_), 2.75 (s, 3H, C*H*_3,Pic_), 2.57 (s, 4H, C*H*_2,Succ_), 1.96 (s, 3H, OCOC*H*_3_) ppm. ^13^C NMR (126 MHz, DMSO-*d*_6_) δ 178.0 (*C*ONH_Ca_), 177.8 (2x*C*O_Succ_ + O*C*OCH_3_), 161.5 (*C*H_Pic_), 151.7 (*C*H_Pic_), 141.2 (*C*H_Pic_), 128.8 (*C*H_Pic_), 123.9 (*C*H_Pic_), 40.1–39.0 (*C*H_2_N(CO)_2_, under DMSO peak), 38.2 (*C*H_2_NH_Ca_), 28.1 (2x*C*H_2,Succ_), 23.0 (OCO*C*H_3_), 22.9 (*C*H_3,Pic_) ppm. MS (*m*/*z*): calcd C_15_H_22_Cl_2_N_4_O_6_Pt (M + H)^+^, 621.06; found, 621.10. EA calcd for C_15_H_22_Cl_2_N_4_O_6_Pt·0.5H_2_O: C, 28.63; H, 3.68; N, 8.9; found: C, 28.32; H, 3.67; N, 8.82.

#### (OC-6-21)[Acetato(ammine)(cyclobutane-1,1-dicarboxylato)(1-(2-isocyanatoethyl)pyrrolidine-2,5-dione)(2-methylpyridine)platinum(IV)] (PicoCarbo-Succ)

Synthesized according to general procedure **C**. The crude product was purified via preparative RP-HPLC (11% MeCN (containing 0.1% CF_3_COOH) in H_2_O (containing 0.1% CF_3_COOH); isocratic) and lyophilized to obtain the title compound as a white solid (29.0 mg, 61%). ^1^H NMR (500 MHz, DMSO-*d*_6_, major isomer A, minor isomer B) δ 8.53–8.43 (d, *J* = 6.4 Hz, 1H, C*H*_Pic_, B), 8.39–8.32 (d, *J* = 5.8 Hz, 1H, C*H*_Pic_, A), 8.14–8.06 (m, 2H, C*H*_Pic_, A + B), 7.63–7.52 (m, 4H, C*H*_Pic_, A + B), 7.34–6.96 (m, 6H, N*H*_3_, A + B), 6.74–6.64 + 6.17–6.07 (m, 2H, N*H*_Ca_, A + B), 3.49–3.45 (m, 2H, C*H*N(CO)_2_, A + B), 3.30–3.23 (m, 2H, C*H*N(CO)_2_, A + B), 3.18–2.94 (m, 4H, C*H*NH_Ca_, A + B), 2.86 (s, 3H, C*H*_3,Pic_, B), 2.74 (s, 3H, C*H*_3,Pic_, A), 2.64–2.53 (m, 8H, C*H*_2,Succ_, A + B), 2.53–2.45 (m, 3H, C*H*_Carbo_, A + B, under DMSO peak), 2.44–2.35 (m, 3H, C*H*_Carbo_, A + B), 1.99 (s, 3H, OCOC*H*_3_, B), 1.91 (s, 3H, OCOC*H*_3_, A), 1.88–1.62 (m, 6H, C*H*_Carbo_, A + B) ppm. ^13^C NMR (126 MHz, DMSO-*d*_*6*_) δ 177.8 (2x*C*O_Succ_, A + B), 177.8 (*C*ONH_Ca_, A), 177.7 (*C*ONH_Ca_, B), 176.3 (O*C*OCH_3_, B), 175.8 (O*C*OCH_3_, A), 175.6 (2x*C*O_Carbo_, A), 175.2 (2x*C*O_Carbo_, B), 163.3 (*C*_q,Pic_, B), 161.5 (*C*_q,Pic_, A), 151.8 (*C*H_Pic_, A), 150.9 (*C*H_Pic_, B), 142.0 (*C*H_Pic_, B), 141.5 (*C*H_Pic_, A), 129.4 + 128.7 + 124.3 + 124.0 (4x*C*H_Pic_, A + B), 55.4 (*C*H_2,Carbo_, A), 54.7 (*C*H_2,Carbo_, A), 38.5 (*C*H_2_N(CO)_2_, A + B), 38.0 (*C*H_2_NH_Ca_, A + B), 31.9 (2x*C*H_2,Carbo_, A + B), 30.0 (2x*C*H_2,Carbo_, A + B), 28.0 (4x*C*H_Succ_, A + B), 22.3 (*C*H_3,Pic_, A), 22.2 (OCO*C*H_3_, A), 21.9 (*C*H_3,Pic_, B), 21.8 (OCO*C*H_3_, B), 15.6 + 15.1 (2x*C*H_2,Carbo_, A + B) ppm. MS (*m*/*z*): calcd C_21_H_28_N_4_O_6_NaPt (M + Na)^+^, 714.13; found, 714.18. EA calcd C_21_H_28_N_4_O_10_Pt·0.5TFA·0.5H_2_O: C, 34.88; H, 3.89; N, 7.4; found: C, 34.69; H, 3.87; N, 7.7.

#### (OC-6-21)[Acetato(ammine)(1-(2-isocyanatoethyl)pyrrolidine-2,5-dione)(2-methylpyridine)(oxalato)platinum(IV)] (PicoOxali-Succ)

Synthesized according to general procedure **C**. The crude product was purified via preparative RP-HPLC (7% MeCN (containing 0.1% CF_3_COOH) in H_2_O (containing 0.1% CF_3_COOH); isocratic) and lyophilized to obtain the title compound as a white solid (83.4 mg, 77%). ^1^H NMR (500 MHz, DMSO-*d*_6_) δ 8.56–8.34 (m, 1H, C*H*_Pic_), 8.19–8.13 (t, *J* = 7.6 Hz, 1H, C*H*_Pic_), 7.68–7.63 (d, *J* = 6.4 Hz, 1H, C*H*_Pic_), 7.63–7.57 (t, *J* = 7.9 Hz, 1H, C*H*_Pic_), 7.42–7.06 (bs, 3H, N*H*_3_), 6.89–6.81 + 6.24–6.34 (m, 1H, N*H*_Ca_), 3.49–3.41 (m, 1H, C*H*N(CO)_2_), 3.30–3.23 (m, 1H, C*H*N(CO)_2_), 3.15–3.07 (m, 1H, C*H*NH_Ca_), 3.00–2.92 (m, 1H, C*H*NH_Ca_), 2.77 (s, 3H, C*H*_3,Pic_), 2.56 (s, 4H, C*H*_2,Succ_), 1.94 (s, 3H, OCOC*H*_3_) ppm. ^13^C NMR (126 MHz, DMSO-*d*_6_) δ 177.8 (2x*C*O_Succ_), 176.0 (O*C*OCH_3_), 163.7 (*C*O_Ox_), 163.0 (*C*ONH_Ca_), 162.7 (*C*O_Ox_), 161.6 (*C*H_Pic_), 151.9 (*C*H_Pic_), 141.9 (*C*H_Pic_), 128.9 (*C*H_Pic_), 124.6 (*C*H_Pic_), 38.3 (*C*H_2_N(CO)_2_), 38.1 (*C*H_2_NH_Ca_), 28.0 (2x*C*H_2,Succ_), 22.2 (OCO*C*H_3_), 22.1 (*C*H_3,Pic_) ppm. MS (*m*/*z*): calcd C_17_H_22_N_4_O_10_NaPt (M + Na)^+^, 660.09; found, 660.15. EA calcd for C_17_H_22_N_4_O_10_Pt·0.5TFA·0.5H_2_O: C, 30.34; H, 3.47; N, 7.86; found: C, 30.19; H, 3.51; N, 8.22.

#### (OC-6-21)[Acetato(ammine)dichlorido(cyclobutane-1,1-dicarboxylato)(1-(2-isocyanatoethyl)-1*H*-pyrrole-2,5-dione)(2-methylpyridine)platinum(IV)] (Pico-Mal)

Synthesized according to general procedure **C**. The crude product was purified via preparative RP-HPLC (19% MeCN (containing 0.1% CF_3_COOH) in H_2_O (containing 0.1% CF_3_COOH); isocratic) and lyophilized to obtain the title compound as a yellow-white solid (301.1 mg, 71%). ^1^H NMR (500 MHz, DMSO-*d*_*6*_) δ 8.77–8.63 (m, 1H, C*H*_Pic_), 8.09–8.06 (t, *J* = 7.4 Hz, 1H, C*H*_Pic_), 7.57–7.52 (m, 5H, C*H*_Pic_ + N*H*_3_), 6.99 (s, 2H, C*H*_Mal_), 6.93–6.86 + 6.27–6.37 (m, 1H, N*H*_Ca_), 3.49–3.45 (m, 1H, C*H*N(CO)_2_), 3.36–3.32 (m, 1H, C*H*N(CO)_2_), 3.21–3.17 (m, 1H, C*H*NH_Ca_), 2.94–2.89 (m, 1H, C*H*NH_Ca_), 2.74 (s, 3H, C*H*_3,Pic_), 1.95 (s, 3H, OCOC*H*_3_) ppm. ^13^C NMR (126 MHz, DMSO-*d*_6_) δ 177.6 (O*C*OCH_3_, ppm value taken from [^1^H–^13^C] HMBC cross-peak), 171.1 (2x*C*O_Mal_), 163.9 (*C*ONH_Ca_), 161.5 (*C*H_Pic_), 151.7 (*C*H_Pic_), 141.2 (*C*H_Pic_), 134.5 (2x*C*H_Mal_), 128.8 (*C*H_Pic_), 123.9 (*C*H_Pic_), 40.1–39.0 (*C*H_2_N(CO)_2_, under DMSO peak), 37.7 (*C*H_2_NH_Ca_), 23.0 (*C*H_3,Pic_), 22.9 (OCO*C*H_3_) ppm. MS (*m*/*z*): calcd C_15_H_21_Cl_2_N_4_O_6_Pt (M + H)^+^, 619.05; found, 619.11. EA calcd for C_15_H_20_Cl_2_N_4_O_6_Pt·0.5TFA·0.5H_2_O: C, 28.08; H, 3.17; N, 8.19; found: C, 28.07; H, 3.44; N, 8.41.

#### (OC-6-21)[Acetato(ammine)(cyclobutane-1,1-dicarboxylato)(1-(2-isocyanatoethyl)-1*H*-pyrrole-2,5-dione)(2-methylpyridine)platinum(IV)] (PicoCarbo-Mal)

Synthesized according to general procedure **C**. The crude product was purified via preparative RP-HPLC (14% MeCN (containing 0.1% CF_3_COOH) in H_2_O (containing 0.1% CF_3_COOH); isocratic) and lyophilized to obtain the title compound as a white solid (234.5 mg, 77%). ^1^H NMR (500 MHz, DMSO-*d*_6_, major isomer A, minor isomer B) δ 8.48–8.47 (d, *J* = 6.3 Hz, 1H, C*H*_Pic_, A), 8.36–8.35 (d, *J* = 5.8 Hz, 1H, C*H*_Pic_, B), 8.13–8.07 (m, 2H, C*H*_Pic_, A + B), 7.60–7.54 (m, 4H, C*H*_Pic_, A + B), 7.23–7.14 (m, 6H, N*H*_3_, A + B), 6.98 (s, 4H, C*H*_Mal_, A + B), 6.79–6.75 + 6.23–6.16 (m, 2H, N*H*_Ca_, A + B), 3.48–3.41 (m, 2H, C*H*N(CO)_2_, A + B), 3.34–3.31 (m, 2H, C*H*N(CO)_2_, A + B), 3.21–3.16 (m, 1H, C*H*NH_Ca_, B), 3.13–3.02 (m, 2H, C*H*NH_Ca_, A), 2.95–2.90 (m, 1H, C*H*NH_Ca_, B), 2.87 (s, 3H, C*H*_3,Pic_, A), 2.74 (s, 3H, C*H*_3,Pic_, B), 2.57–2.54 (m, 3H, C*H*_Carbo_, A + B), 2.53–2.45 (m, 2H, C*H*_Carbo_, A + B), 2.41–2.32 (m, 3H, C*H*_Carbo_, A + B), 1.99 (s, 3H, OCOC*H*_3_, A), 1.91 (s, 3H, OCOC*H*_3_, B), 1.85–1.76 (m, 2H, C*H*_Carbo_, A), 1.71–1.65 (m, 2H, C*H*_Carbo_, B) ppm. ^13^C NMR (126 MHz, DMSO-*d*_*6*_) δ 176.7 (O*C*OCH_3_, A, ppm value taken from [^1^H–^13^C] HMBC cross-peak), 176.3 + 175.8 + 175.5 (4x*C*O_Carbo_, A + B), 175.2 (O*C*OCH_3_), B), 171.1 + 171.0 (2x*C*O_Mal_, A + B), 163.3 + 163.2 (2x*C*ONH_Ca_, A + B), 162.9 (*C*H_Pic_, A), 161.5 (*C*H_Pic_, B), 151.7 (*C*H_Pic_, A), 150.9 (*C*H_Pic_, B), 142.0 + 141.5 (2x*C*H_Pic_, A + B), 134.5 (2x*C*H_Mal_, A + B), 129.4 + 128.7 + 124.3 + 124.0 (4x*C*H_Pic_, A + B), 55.3 + 54.7 (2x*C*H_2,Carbo_, A + B), 40.1–39.0 (2x*C*H_2_N(CO)_2_, under DMSO peak), 37.6 + 37.5 (2x*C*H_2_NH_Ca_, A + B), 32.1 + 30.7 + 29.8 + 29.5 (4x*C*H_2,Carbo_, A + B), 22.3 (OCO*C*H_3_, B), 22.2 (*C*H_3,Pic_, B), 21.9 (OCO*C*H_3_, A), 21.8 (*C*H_3,Pic_, A), 15.6 + 15.1 (2x*C*H_2,Carbo_, A + B) ppm. MS (*m*/*z*): calcd. C_21_H_26_N_4_O_10_NaPt (M + Na)^+^, 712.12; found, 712.15. EA calcd for C_21_H_26_N_4_O_10_Pt·1TFA: C, 34.38; H, 3.39; N, 6.97; found: C, 34.43; H, 3.22; N, 7.23.

#### (OC-6-21)[Acetato(ammine)(1-(2-isocyanatoethyl)-1*H*-pyrrole-2,5-dione)(2-methylpyridine)(oxalato)platinum(IV)] (PicoOxali-Mal)

Synthesized according to general procedure **C**. The crude product was purified via preparative RP-HPLC (12% MeOH (containing 0.1% CF_3_COOH) in H_2_O (containing 0.1% CF_3_COOH); isocratic) and lyophilized to obtain the title compound as a white solid (28.7 mg, 70%). ^1^H NMR (500 MHz, DMSO-*d*_6_) δ 8.39–8.38 (m, 1H, C*H*_Pic_), 8.18–8.15 (t, *J* = 7.6 Hz, 1H, C*H*_Pic_), 7.67–7.65 (d, *J* = 7.4 Hz, 1H, C*H*_Pic_), 7.63–7.61 (t, *J* = 6.5 Hz, 1H, C*H*_Pic_), 7.25 (b, 3H, N*H*_3_), 6.98 (s, 2H, C*H*_Mal_), 6.95–6.93 + 6.40–6.26 (m, 1H, N*H*_Ca_), 3.48–3.44 (m, 1H, C*H*N(CO)_2_), 3.35–3.30 (m, 1H, C*H*N(CO)_2_), 3.19–3.13 (m, 1H, C*H*NH_Ca_*)*, 2.92–2.87 (m, 1H, C*H*NH_Ca_), 2.76 (s, 3H, C*H*_3,Pic_), 1.94 (s, 3H, OCOC*H*_3_) ppm. ^13^C NMR (126 MHz, DMSO-*d*_*6*_) δ 175.9 (O*C*OCH_3_), 171.0 (2x*C*O_Mal_), 163.7 (*C*ONH_Ca_), 163.1 (*C*O_Ox_), 162.7 (*C*H_Pic_), 161.6 (*C*O_Ox_), 151.9 (*C*H_Pic_), 141.9 (*C*H_Pic_), 134.4 (2x*C*H_Mal_), 128.9 (*C*H_Pic_), 124.6 (*C*H_Pic_), 40.1–39.0 (*C*H_2_N(CO)_2_, under DMSO peak), 37.5 (*C*H_2_NH_Ca_), 22.2 (*C*H_3,Pic_), 22.1 (OCO*C*H_3_) ppm. MS (*m*/*z*): calcd. C_17_H_20_N_4_O_10_NaPt (M + Na)^+^, 658.07; found, 658.12. EA calcd for C_17_H_20_N_4_O_10_Pt·1TFA: C, 30.45; H, 2.82; N, 7.48; found: C, 30.37; H, 2.93; N, 7.87.

#### (OC-6-44)[Acetatodiamminedichlorido[2-(2,5-dioxopyrrolidin-1-yl)butyl)carbamato]platinum(IV)] (Cis-Succ)

Cis-OH-OAc^41^ and 1-(4-isocyanatobutyl)pyrrolidine-2,5-dione^41^ were synthesized, as described previously. Cis-OH-OAc (127.9 mg, 0.61 mmol)) was dissolved in anhydrous DMF (4 mL) under an argon atmosphere at RT. 1-(4-Isocyanatobutyl)pyrrolidine-2,5-dione was added, and the resulting mixture was stirred at RT in the dark for 23 h. The crude product was purified via preparative RP-HPLC (20% MeCN (containing 0.1% HCOOH) in H_2_O (containing 0.1% CHCOOH); isocratic) and lyophilized to obtain the title compound as a white solid (23.6 mg, 14%). ^1^H NMR (500 MHz, DMSO-*d*_6_) δ 6.77–6.41 (m, 7H, N*H*_3_ + N*H*_Ca_), 3.31–3.25 (m, 2H, C*H*_2_N(CO)_2_, under H_2_O peak), 2.83–2.77 (m, 2H, C*H*_2_NH_Ca_, 2H), 2.07 (s, 4H, C*H*_2,Succ_), 1.82 (s, 3H, OCOC*H*_3_), 1.45–1.18 (m, 6H, C*H*_2_) ppm. MS (*m*/*z*): calcd C_12_H_24_Cl_2_N_4_O_6_NaPt (M + Na)^+^, 609.32; found, 609.06.

### Stability and Reduction Experiments

#### HPLC Experiments–Reduction with AA

PB (150 mM, pH 7.4) containing 1 mM Pt compound was incubated at 20 °C with and without the addition of 10 equiv l-ascorbic acid or reduced GSH. The reaction was monitored on a Thermo Scientific Dionex UltiMate 3000 UHPLC-system using a Waters Acquity UPLC BEH C18 1.7 μm, 3.0 × 50 mm column. Milli-Q water containing 0.1% formic acid and acetonitrile containing 0.1% formic acid were used as eluents. A gradient of 5–95% over 5 min was used with a flow of 0.6 mL/min. To evaluate the reaction process, we used the peak area of the parental complex. This was done because in most cases, the reduction products did not have a sufficient retention time to be distinguished from the injection peak.

#### HPLC Experiments–Reduction with CytC or AA

PB (50 mM, pH 6.0) containing 700 μM Pt compound was incubated at 37 °C with the addition of 0.1 equiv horse heart CytC and 10 equiv reduced NADH or with 10 equiv l-ascorbic acid. The reaction was monitored on a Thermo Scientific Dionex UltiMate 3000 UHPLC-system using a Waters Acquity UPLC BEH peptide C18 1.7 μm 2.1 × 100 mm column. Milli-Q water, containing 0.1% formic acid, and acetonitrile containing 0.1% formic acid were used as eluents. A gradient of 5–95% over 10 min was used with a flow of 0.4 mL/min. To evaluate the reaction process, the peak area of the parental complex was used. This was done due to the fact that in most cases, the reduction products did not have a sufficient retention time to be distinguished from the injection peak.

### NMR Experiments

An 1 mM solution of Pt compound was incubated in a mixture of 90% PB or PBS (150 mM, pH 7.4) and 10% D_2_O at 37 °C with and without the addition of 10 equiv GSH. The reaction was monitored with ^1^H or [^1^H, ^15^N] HSQC (for ^15^N-cisplatin) NMR spectroscopy using water suppression, recorded on a Bruker AV Neo 500 spectrometer at 298 K. For ^1^H NMR spectra, superimposed spectral overlay was used to enable relative quantification of the integrations of the C*H*_3,2-Pic_ signal of the parental complex at single time points (2, 4, 6, or 8 h) compared to the integration of the reference C*H*_3,2-Pic_ signal at time point 0 h. The reference spectrum was shifted with a value of −0.5 ppm compared to the original ppm value calibrated to D_2_O before overlay with a spectrum at a single time point. In case of ^15^N-cisplatin, the cross-peak of the parental complex in [^1^H, ^15^N] HSQC at time point 0 h was used as reference, and spectral overlay was achieved by shifting the 2D spectra at all given time points (2, 4, 6, and 8 h) with vertical steps of +14 ppm compared to the original ^15^N NMR ppm value of the parental complex.

### SEC-ICP-MS Measurements

FCS was purchased from Sigma-Aldrich and buffered with 150 mM phosphate pH 7.4 in order to guarantee a stable pH. The platinum(IV) complexes were dissolved in 150 mM PB (pH 7.4) at a concentration of 5 mM and diluted 1:100 in buffered serum to obtain a final concentration of 50 μM. The samples were then incubated in the autosampler at 37 °C and analyzed at 0, 1, 4, 24, and 25 h time points. Between each sample, a pure water blank was measured. For SEC-ICP-MS measurements, an Agilent 1260 Infinity system coupled to an Agilent 7800 ICP-MS system equipped with a dynamic reaction cell was used. Oxygen (purity 5.5, Messer Austria GmbH, Gumpoldskirchen, Austria) was used as the reaction gas. HPLC parameters are given in Table S1, and ICP-MS operation parameters are given in Table S2.

### Biological Analysis

#### Cell Culture

For the cell culture assays, the human ovarian model A2780/wt and its corresponding cisplatin-resistant subline A2780/cis (both purchased from Sigma and cultured in RPMI1640 medium), the human colorectal cancer model HCT116 (cultured in McCoy′s medium), and the murine (Balb/c) colorectal cancer model CT26 (cultured in DMEM/F-12 (1:1) medium) were used. The colon cancer models were purchased from the American Type Culture Collection (ATCC). The media were supplemented with 10% FCS (purchased from PAA Linz, Austria). To maintain drug resistance of A2780/cis, the subline was cultivated under continuous selection pressure (1 μM cisplatin). The selection drug was removed 1 week before the experiments were performed. Cell cultures were maintained at 37 °C in humidified atmosphere containing 5% CO_2_. All cultures were free of antibiotics and regularly checked for mycoplasma contamination. All cell culture media and reagents were purchased from Sigma-Aldrich Austria. All investigated drugs were dissolved in either water (oxaliplatin, carboplatin) or DMF before further dilution in cell culture medium. DMF concentrations were always below 0.5%.

#### Cell Viability by MTT-Based Assay (72 h)

Cells were plated in 96-well plates ((2–4) × 10^4^ cells in 100 μL of well) and left to recover for 24 h. Subsequently, the cells were incubated with the tested compounds in rising concentrations. After 72 h incubation, cell viability was analyzed using an MTT-based assay (EZ4U, Biomedica, Austria) following the manufacturer’s recommendations. The received data were visualized as dose–response curves by Graph Pad Prism 5 software (Graph Pad software, USA).

#### Cell Viability By Colony Formation Assay (7 Days)

Cells were plated in 24-well plates (250–1000 cells in 500 μL of growth medium per well) and left to recover for 24 h in the incubator. Then, cells were exposed to the indicated drugs in rising concentrations. After 7 days, the cells were fixed with methanol for 20 min at 4 °C and stained with crystal violet 5 (0.01% in PBS). The fluorescence of the crystal violet was detected by the Typhoon scanner (Typhoon TRIO Variable Mode Imager, GE Healthcare Life Sciences) after excitation by the red laser (633 nm). Quantification was done with ImageJ software.

#### Animals

Ten-week-old Balb/c mice were purchased from Envigo Laboratories (San Pietro al Natisone, Italy). The animals were kept in pathogen-free conditions in a controlled environment with 12 h light–dark cycle, and every procedure was done in a laminar airflow cabinet. Animals were controlled for distress development every day, and tumor size was assessed regularly by caliper measurement. Tumor volume was calculated using the formula: (length × width^2^)/2. All experiments were approved by the Ethics Committee for the Care and Use of Laboratory Animals at the Medical University Vienna (BMWF-2022-0.770.291) and performed according to the guidelines from the Austrian Animal Science Association and the Federation of European Laboratory Animal Science Associations (FELASA).

#### Serum PK and Tumor/Organ Distribution

CT-26 cells (5 × 10^5^ cells in 50 μL of serum-free medium) were subcutaneously injected into the right flank of male Balb/c mice. Ten days postinjection, the mice received an i.v. administration of the test drugs (*n* = 4–8 per treatment group), with a dose equimolar to 20 mg/kg picoplatin. Picoplatin and the three maleimide-containing platinum(IV) complexes were dissolved in 30% PEG400-containing 0.9% NaCl and PBS (Cytiva, USA), respectively. For PK analysis, blood samples were collected at 5 min, 30 min, 5 h, and 24 h postadministration (2–4 mice per time point) via the facial vein. After a 10 min clotting period of the blood at RT, the serum was separated by centrifugation (900 g, 10 min, repeated twice). Tumor and organ accumulation of the drug was assessed at 5 and 24 h postadministration (2 animals per time point). Mice were euthanized via cervical dislocation, and their tumors and organs were excised. All collected samples were stored at −20 °C until further processing for Pt concentration analysis by ICP-MS. For ICP-MS measurements, nitric acid (67–69%, Suprapur, NORMATOM; VWR International, Austria) and hydrogen peroxide (30%, Suprapur; Merck) were used without further purification. Approximately 15–30 mg of tissue (gravimetrically measured) was digested with 2 mL of 20% nitric acid and 100 μL of H_2_O_2_ in an open-vessel graphite digestion system (Labter, ODLAB; AHF Analysentechnik AG, Germany), using PFA vials and lids. The digested samples were diluted with Milli-Q water (18.2 MΩ·cm, Milli-Q Advantage, Darmstadt, Germany) before Pt concentration determination via ICP-MS. Pt and Rh standards were obtained from CPI International (Amsterdam, The Netherlands). Quantification of total Pt content was performed on a quadrupole-based ICP-MS instrument (Agilent 7800, Agilent Technologies, Tokyo, Japan), equipped with an Agilent SPS 4 autosampler and a MicroMist nebulizer. The sample uptake rate was approximately 0.2 mL/min. Instrument settings included an RF power of 1550 W, with nickel cones and argon as both the plasma gas (15 L/min) and the carrier gas (∼1.1 L/min). The dwell time was set to 0.1 s, and measurements were conducted in 12 replicates with 100 sweeps each. Rh was used as the internal standard for Pt quantification. Data were processed using the Agilent MassHunter Workstation Software (ver. B.01.04, 2018).

#### Anticancer Activity In Vivo

CT26 cells (5 × 10^5^ in serum-free medium) were injected subcutaneously into the right flank of male Balb/c mice. Therapy was started when tumor nodules were palpable (day 3). Animals were treated i.v. with **Pico-Mal** (36.4 mg/kg dissolved in PBS), **PicoCarbo-Mal** (42.7 mg/kg dissolved in PBS), **PicoOxali-Mal** (39.9 mg/kg dissolved in PBS), or an equimolar dose of picoplatin (20 mg/kg dissolved in 30% PEG400-containing 0.9% NaCl) on days 3 and 10 after cell injection. Mice were sacrificed by cervical dislocation in the case of a tumor length >20 mm, tumor ulceration, or other signs for distress.

## Supporting Information Available

Interconversion of PicoCarbo-Succ isomers, stability kinetics of platinum(II) complexes, stability and reduction kinetics of platinum(IV) complexes, sulfur traces of FCS, SEC-ICP-MS operation/flow parameters, SEC-ICP-MS results of picoplatin, anticancer activity and overall survival of carboplatin and oxaliplatin, organ distribution of the platinum complexes, synthesis of cis-Succ, and ^1^H and ^13^C NMR spectra of all final platinum(IV) complexes (PDF)
