# Supplementary material for: Exploring the Structure–Activity Relationships of Albumin-Targeted Picoplatin-Based Platinum(IV) Prodrugs
Source: Inorg Chem. 2025 Jan 29;64(5):2554–66. doi: 10.1021/acs.inorgchem.4c05269 (PMC11815855; doi:10.1021/acs.inorgchem.4c05269)
Supplement: Supplementary file 1 — ic4c05269_si_001.pdf [file ic4c05269_si_001.pdf]

Exploring the Structure-Activity Relationships of Albumin-targeted Picoplatin-based Platinum(IV) Prodrugs

Martijn Dijkstra, ‡[a,b] Hemma Schueffl, ‡[c] Barbora Adamova,[c] Oliver Baumfried,[c] Alexander Kastner,[a] Walter Berger,[c,d] Bernhard K. Keppler,[a,d] Petra Heffeter,[c,d*] and Christian R. Kowol[a,d*]

A) University of Vienna, Faculty of Chemistry, Institute of Inorganic Chemistry, Waehringer Str. 42, 1090 Vienna, Austria. B) University of Vienna, Vienna Doctoral School in Chemistry (DoSChem), Waehringer Str. 42, 1090 Vienna, Austria. C) Center for Cancer Research and Comprehensive Cancer Center, Medical University of Vienna, Borschkegasse 8a, 1090 Vienna, Austria. D) Research Cluster "Translational Cancer Therapy Research", 1090 Vienna, Austria.

*Email: christian.kowol@univie.ac.at;

*Email: petra.heffeter@meduniwien.ac.at.

‡These authors contributed equally to this work; *shared position.

Table of contents

	Content	Page number
Figure S1	Interconversion of isomers of 1 mM PicoCarbo-Succ	2
Figure S2	Platinum(II) stability in 10% D_2O/90% PBS	2
Figure S3	Platinum(II) stability in 10% D_2O/90% PB	3
Figure S4	^1H NMR kinetics of 1:10 mM picoplatin:GSH	4
Figure S5	[^1H, ^{15}N] NMR kinetics of 1:10 mM ^{15}N-cisplatin:GSH	5
Figure S6	Reduction of Pico-Succ with 0, 0.5 or 1 eq. AA	6
Figure S7	Platinum(IV)-Succ stability in PB	6
Figure S8	Reduction of Pico-Succ, PicoCarbo-Succ and PicoOxali-Succ with 10 eq. of GSH	7
Figure S9	Sulfur traces of FCS	7
Figure S10	SEC-ICP-MS results of picoplatin	8
Figure S11	Anticancer activity and overall survival of carboplatin and oxaliplatin against CT-26-bearing Balb/c mice.	9
Figure S12	Organ distribution of the platinum complexes in CT-26-bearing Balb/c mice	10
Table S1	SEC-ICP-MS operation parameters	11
Table S2	SEC-ICP-MS flow injection parameters	11
Scheme S1	Synthesis of Cis-Succ	11
Figure S13-18	NMR characterization of final platinum(IV) complexes	12-17

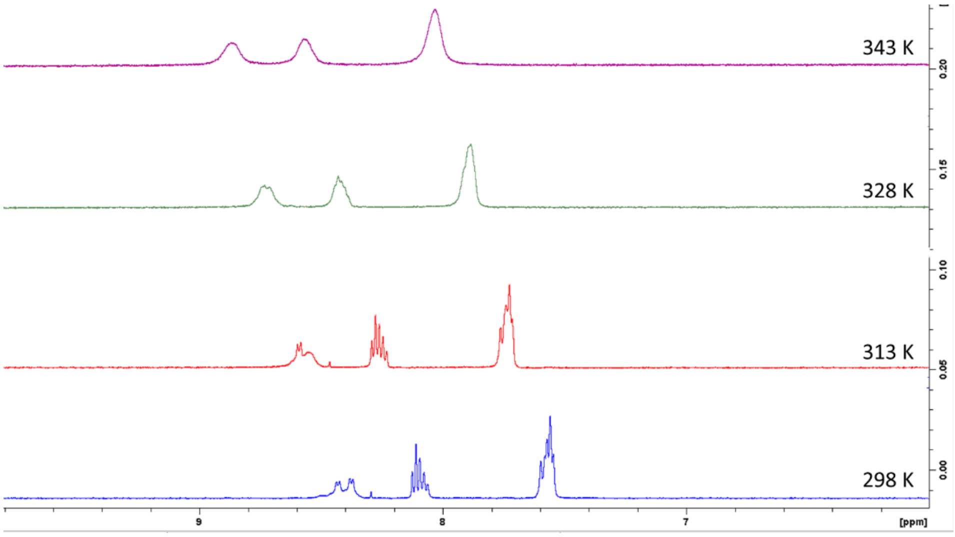

Figure S1. Interconversion of isomers of 1 mM **PicoCarbo-Succ**, analyzed with ^{1}H NMR in DMSO-d_6 at different temperatures.

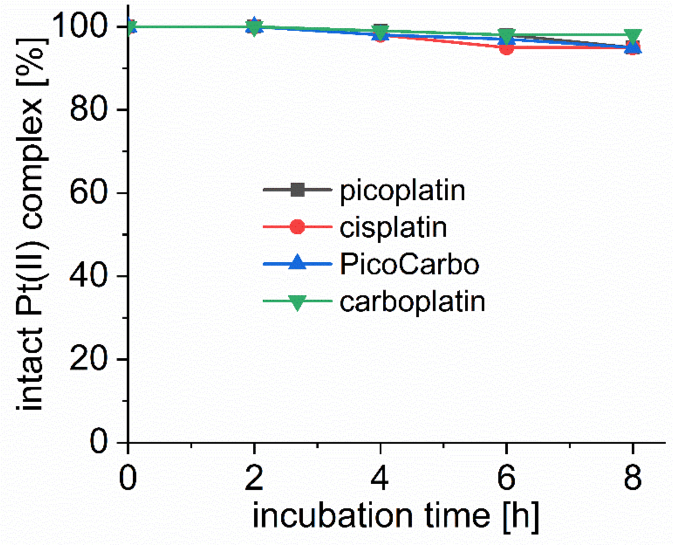

Figure S2. Stability of 1 mM picoplatin, ^{15}N-cisplatin, **PicoCarbo** and carboplatin in 10% D_2O/90% PBS (pH = 7.4) at 37°C over 8 h, analyzed with ^{1}H NMR or [^{1}H, ^{15}N] HSQC NMR.

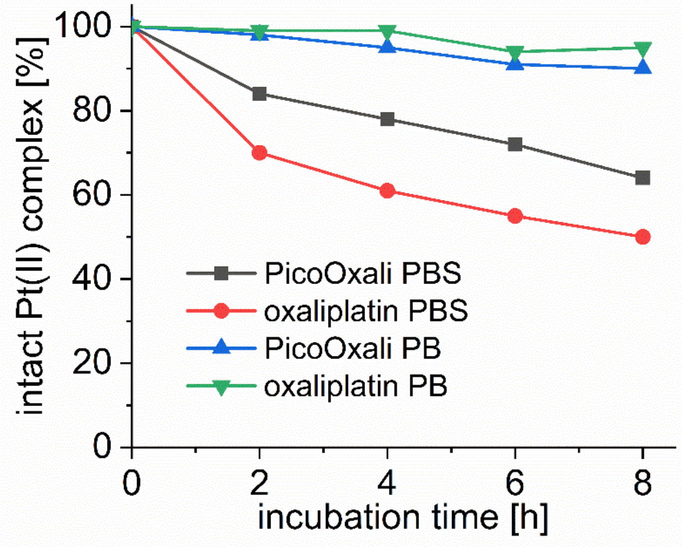

Figure S3. Stability of 1 mM **PicoOxali** and oxaliplatin in 10% D_2O/90% PBS and 10% D_2O/90% PB (pH = 7.4) at 37°C over 8 h, analyzed with ^{1}H NMR.

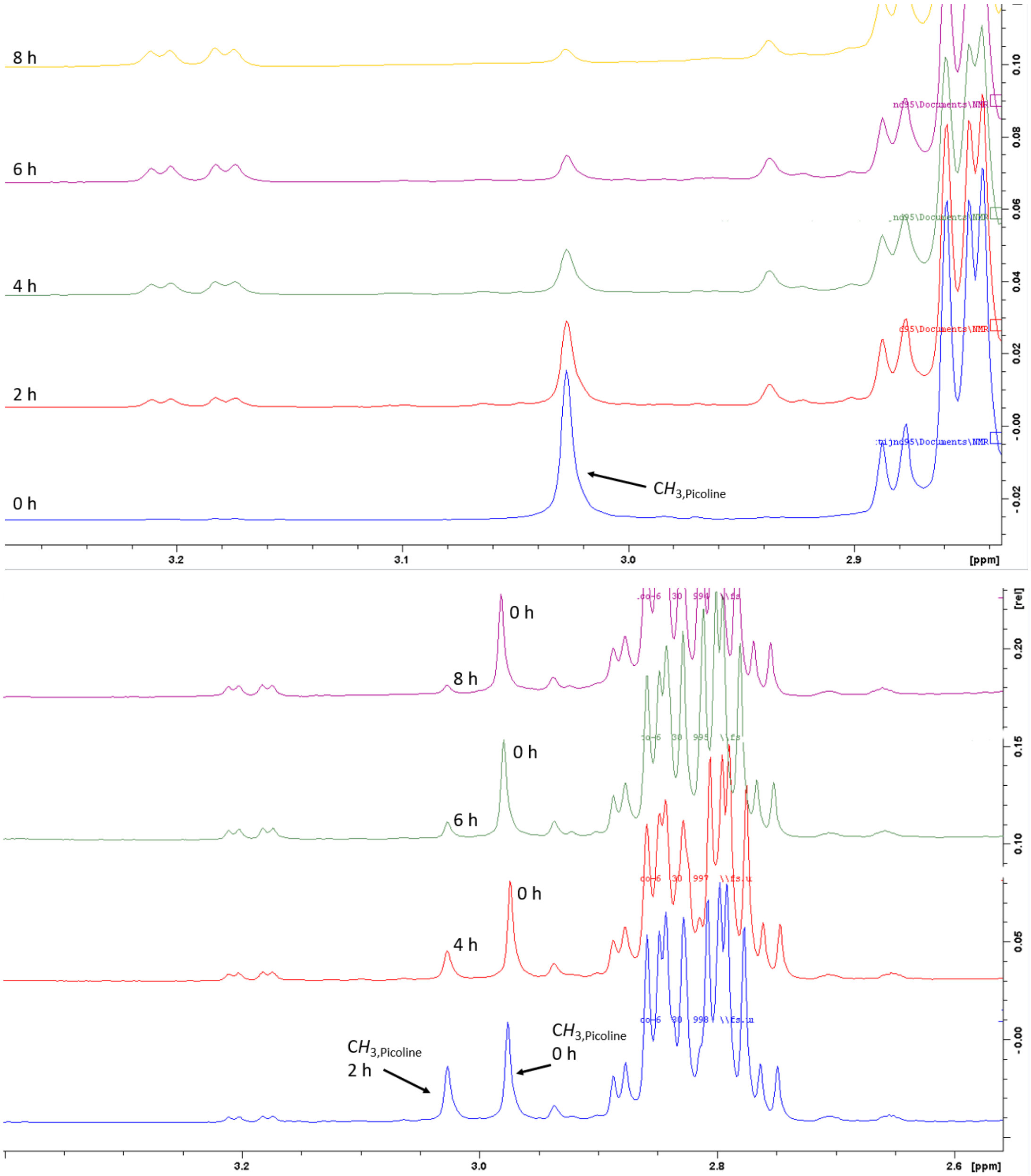

Figure S4. Interaction of 1 mM picoplatin with 10 eq. GSH in 10% D_2O and 90% PB (pH 7.4) at 37°C over 8 h, monitored with ^1H NMR (top). Superimposed spectra (bottom) were used to enable relative quantification of the integrations of the $CH_{3,2\text{-Pic}}$ signal of the parental complex at single timepoints (2, 4, 6 or 8 h) compared to the integration of the reference $CH_{3,2\text{-Pic}}$ signal at timepoint 0 h. The reference spectrum was shifted with a value of -0.5 ppm compared to the original ppm value to enable individual integration.

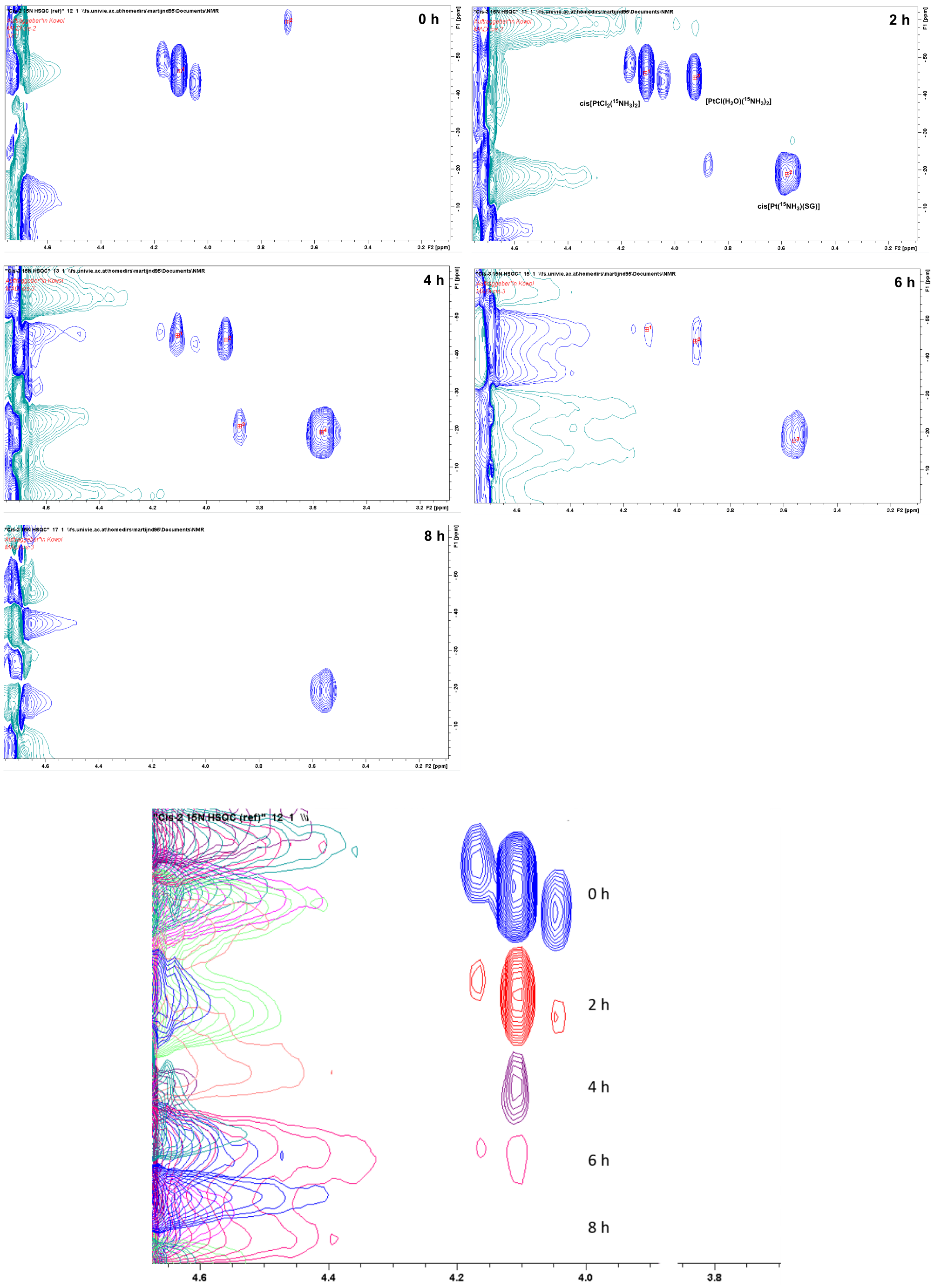

Figure S5. Interaction of 1 mM ^{15}N-cisplatin with 10 eq. GSH in 10% D_2O and 90% PB (pH 7.4) at 37°C over 8h, monitored with [^{1}H, ^{15}N] HSQC NMR (top). Superimposed spectra (bottom) were used to enable relative quantification of the cross-peak integrations of the parental complex at all timepoints (2, 4, 6 or 8 h) compared to the integration of the reference at timepoint 0 h. The spectra at given timepoints were shifted with vertical steps of +14 ppm compared to the original ^{15}N NMR ppm value of the parental complex.

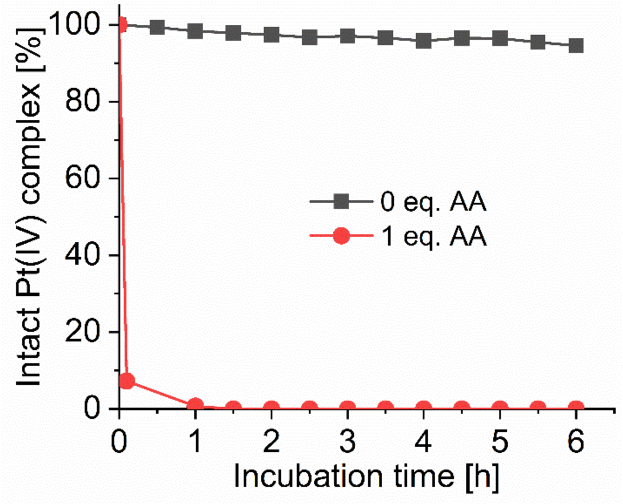

Figure S6. Reduction kinetics of 1 mM **Pico-Succ** in 150 mM PB (pH = 7.4) at 20 °C with 0 or 1 eq. of AA over 6 h, measured with UHPLC.

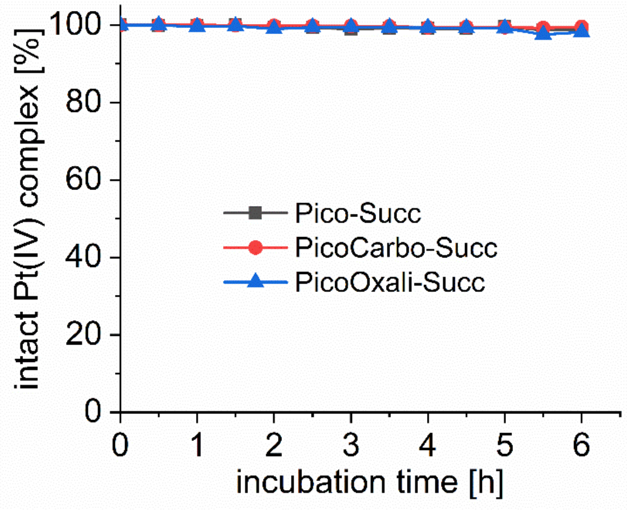

Figure S7. Stability of 1 mM **Pico-Succ**, **PicoCarbo-Succ** and **PicoOxali-Succ** in 150 mM PB (pH = 7.4) at 20 °C over 6 h, measured with UHPLC.

.

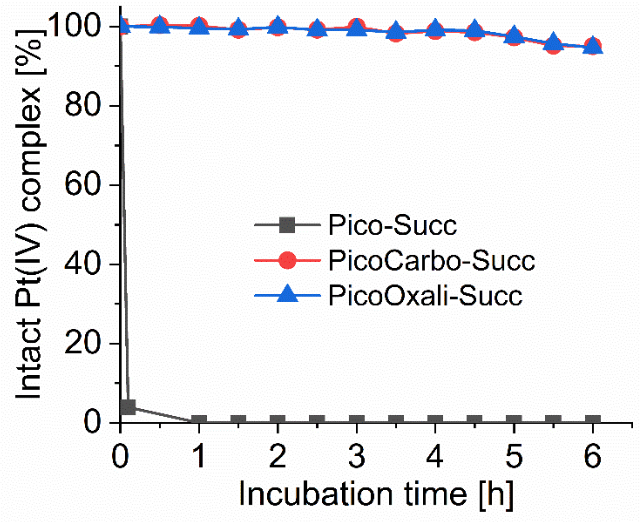

Figure S8. Reduction kinetics of 1 mM **Pico-Succ**, **PicoCarbo-Succ** and **PicoOxali-Succ** in 150 mM PB (pH = 7.4) at 20 °C with 10 eq. of GSH over 6 h, measured with UHPLC.

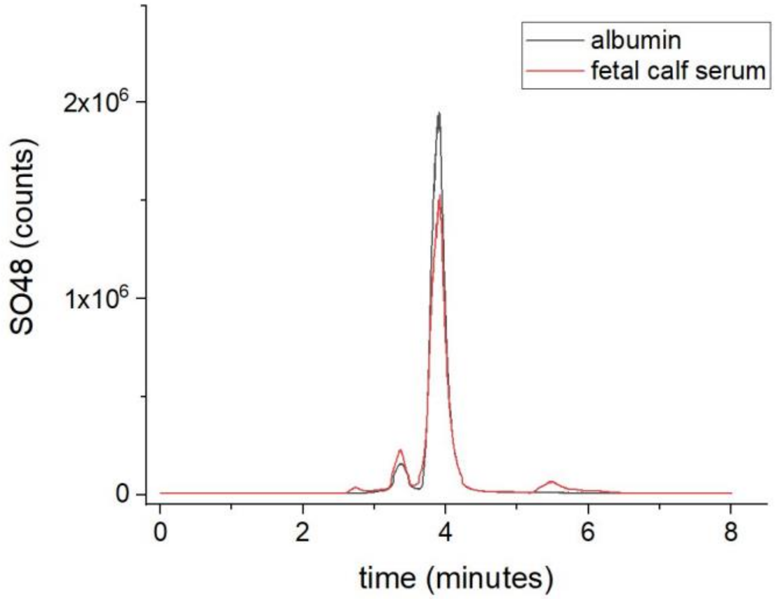

Figure S9. Sulfur traces of FCS (+150 mM PB, pH 7.4) as well as pure albumin in PB (50 mM, pH 7.4), both measured by SEC-ICP-MS. The small peak at ~3.3 min corresponds to the albumin dimer.

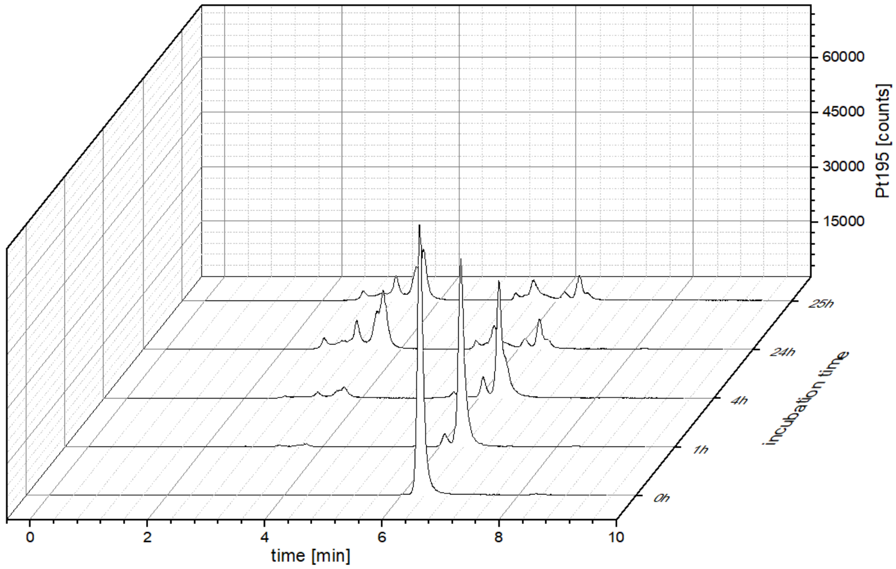

Figure S10. ^{195}Pt-traces of incubation of 50 µM picoplatin in FCS (containing 150 mM PB, pH = 7.4) at 37 °C over 24 h, measured with SEC-ICP-MS.

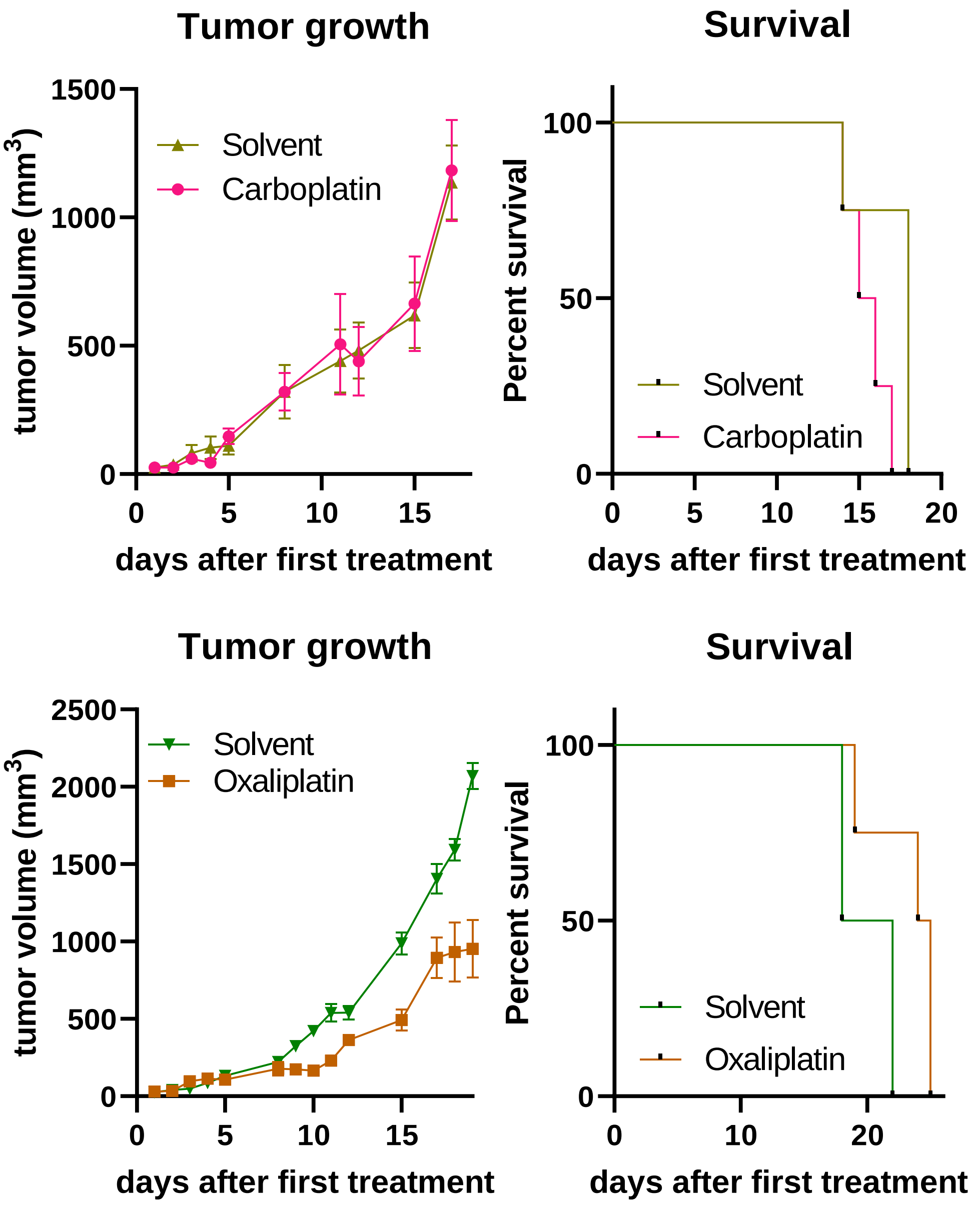

Figure S11: Anticancer activity and overall survival of carboplatin and oxaliplatin against CT-26-bearing Balb/c mice. Mice were treated i.v. with the maximal tolerated dose of the respective drug and compared to the solvent control. Carboplatin (60 mg/kg) was applied once a week for two weeks. Oxaliplatin (9 mg/kg) was applied twice a week for two weeks. Tumor volume data are presented as means ± SEM. The overall survival is depicted via a Kaplan–Meier curve. The oxaliplatin data were used from Schueffl et al, Chem. Sci. *2021*, 12, 12587-12599.

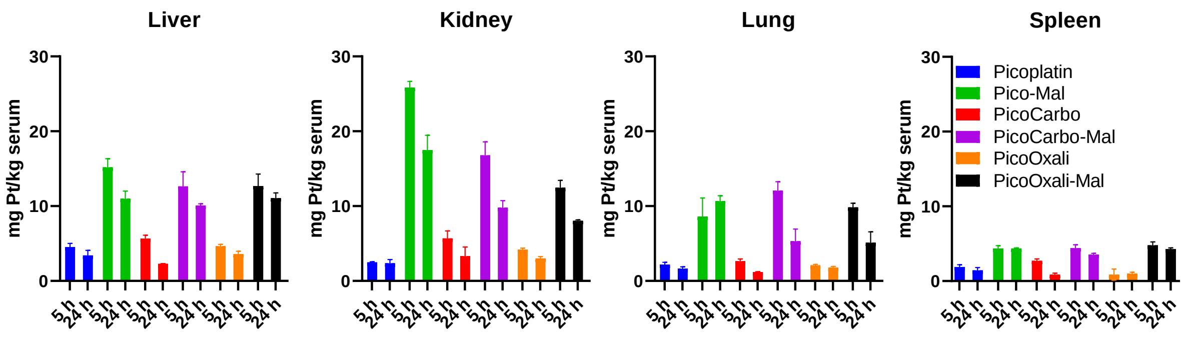

Figure S12: Organ distribution of the platinum complexes in CT-26-bearing Balb/c mice. Animals were treated once i.v. with concentrations equimolar to 20 mg/kg picoplatin. Organ samples were collected after 5 h and 24 h. Pt levels of all samples were measured via ICP-MS. Data given are means ± SEM.

Table S1. HPLC operation parameters used in SEC-ICP-MS measurements

Samples	50 µM in FCS (150 mM PB, pH 7.4)
Column	Acquity UPLC BEH 200Å 1.7 µm, 4.6x150 mm
Eluent	50 mM CH_3COONH_4, pH = 6.8
Flow rate	400 µl/min
Column temperature	37°C
Sample temperature	37°C
Injection volume	5 µl

Table S2 Flow injection parameters used in SEC-ICP-MS measurements

Samples	50 µM in FCS (150 mM PB, pH 7.4)
Column	n.a.
Eluent	50 mM CH_3COONH_4, pH = 6.8
Flow rate	400 µl/min
Column temperature	37°C
Sample temperature	20°C
Injection volume	2 µl

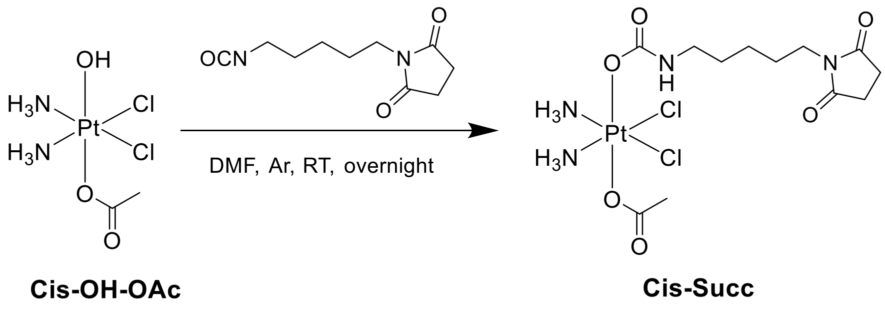

Scheme S1. Synthetic route for **Cis-Succ.**

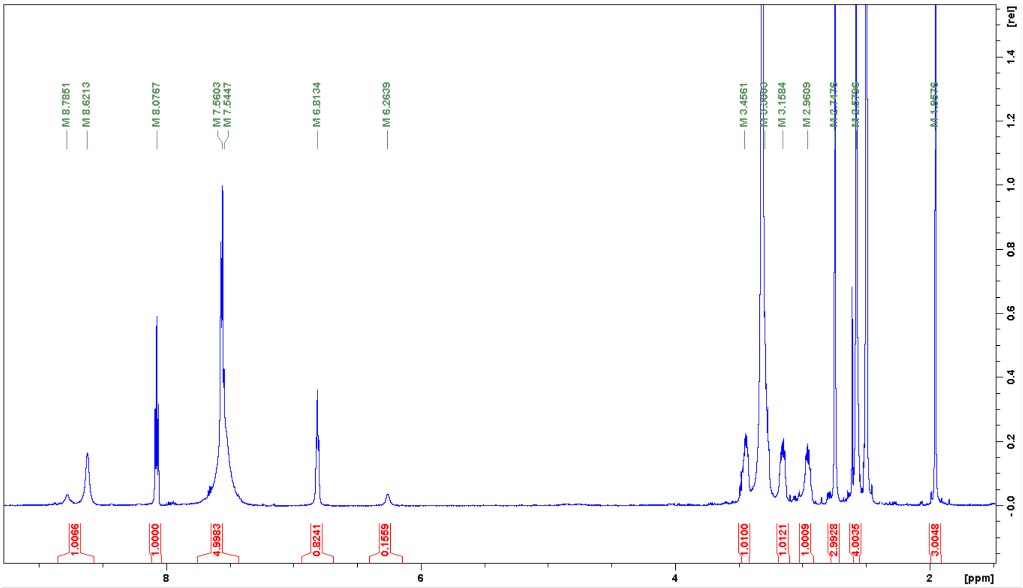

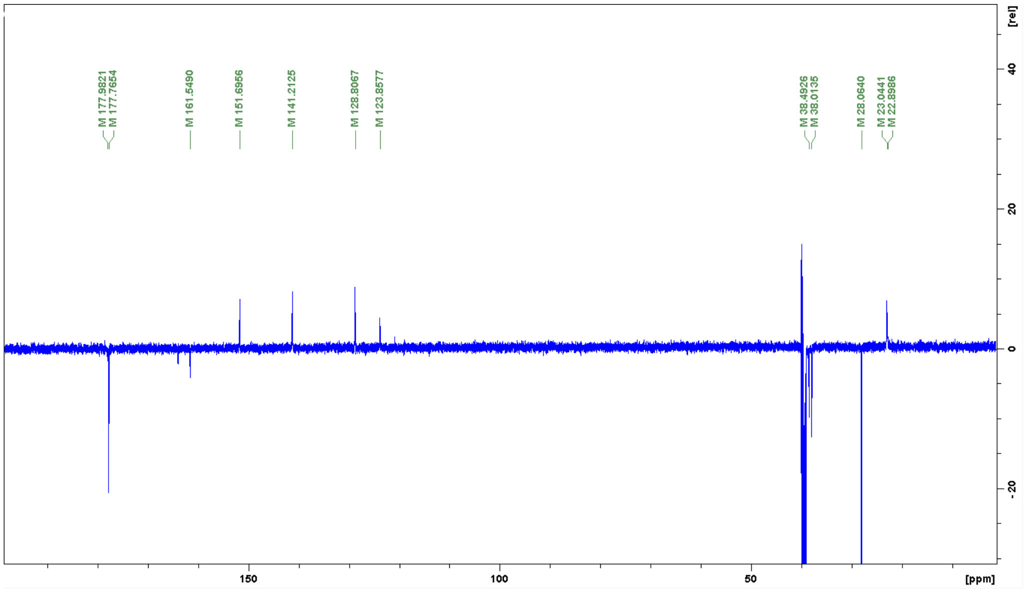

Figure S13. ^{1}H and ^{13}C-NMR spectra of **Pico-Succ**, measured in DMSO-d6.

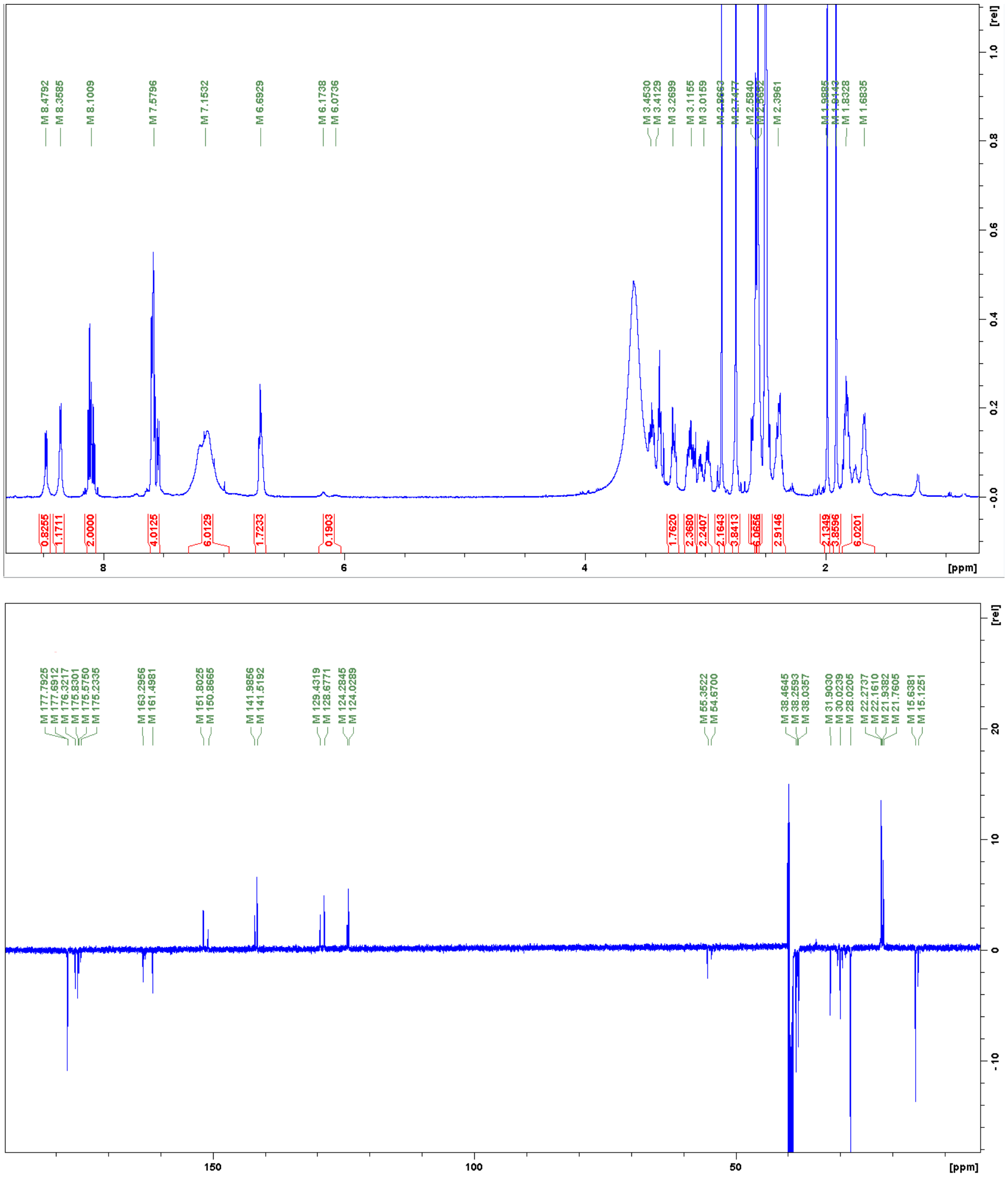

Figure S14. ^{1}H and ^{13}C-NMR spectra of **PicoCarbo-Succ**, measured in DMSO-d6.

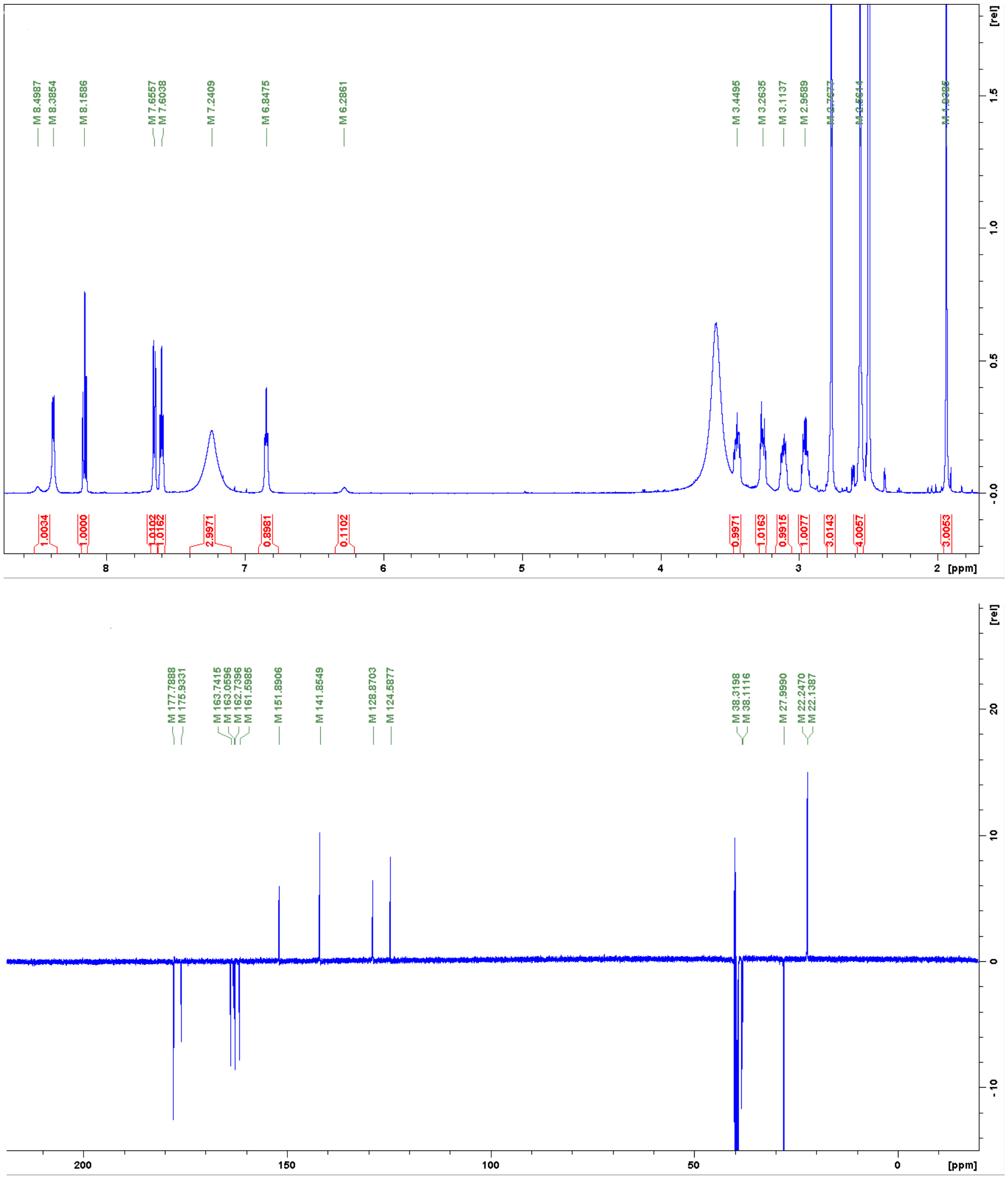

Figure S15. ^{1}H and ^{13}C-NMR spectra of **PicoOxali-Succ**, measured in DMSO-d6.

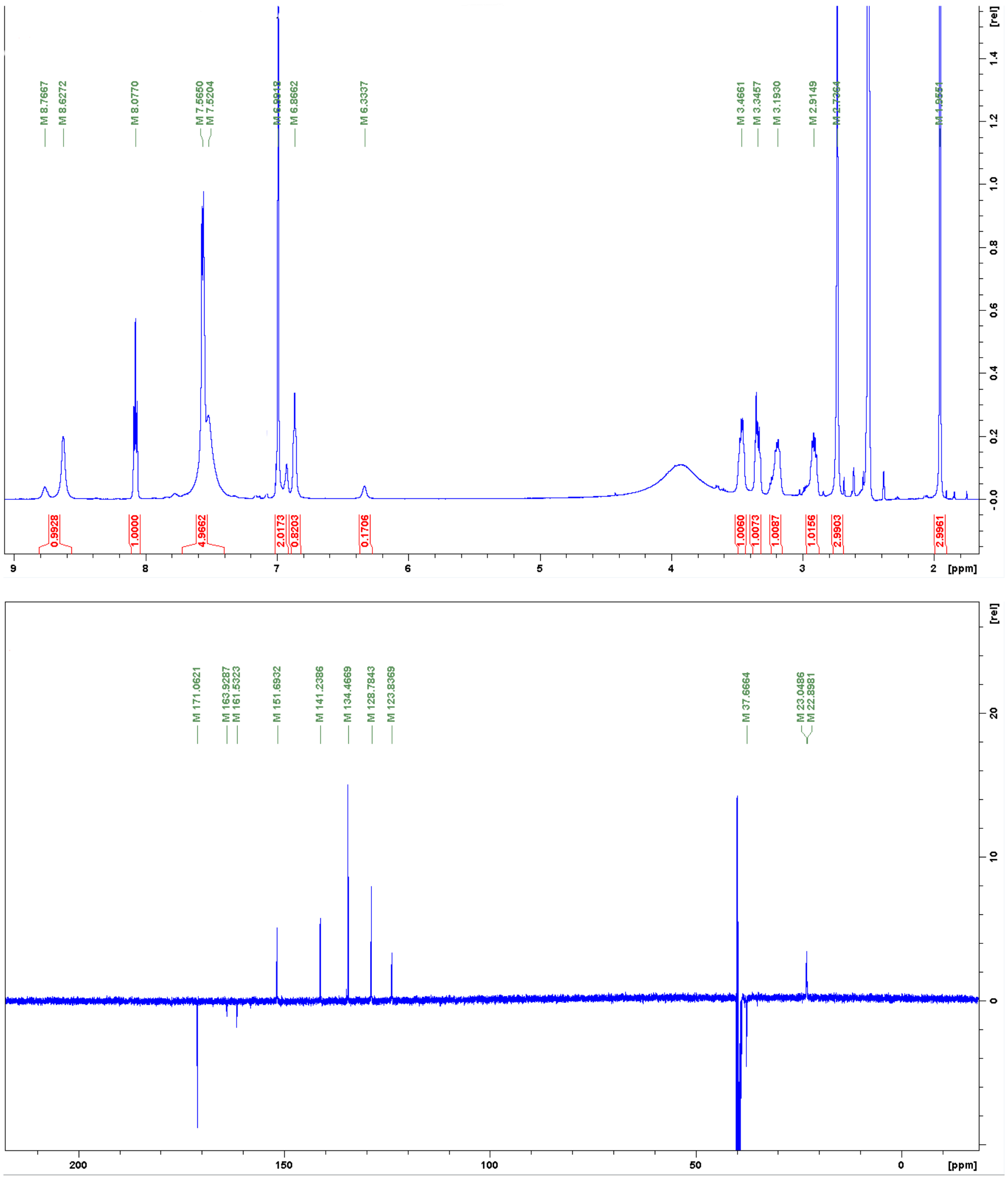

Figure S16. ^{1}H and ^{13}C-NMR spectra of **PicoMal**, measured in DMSO-d6.

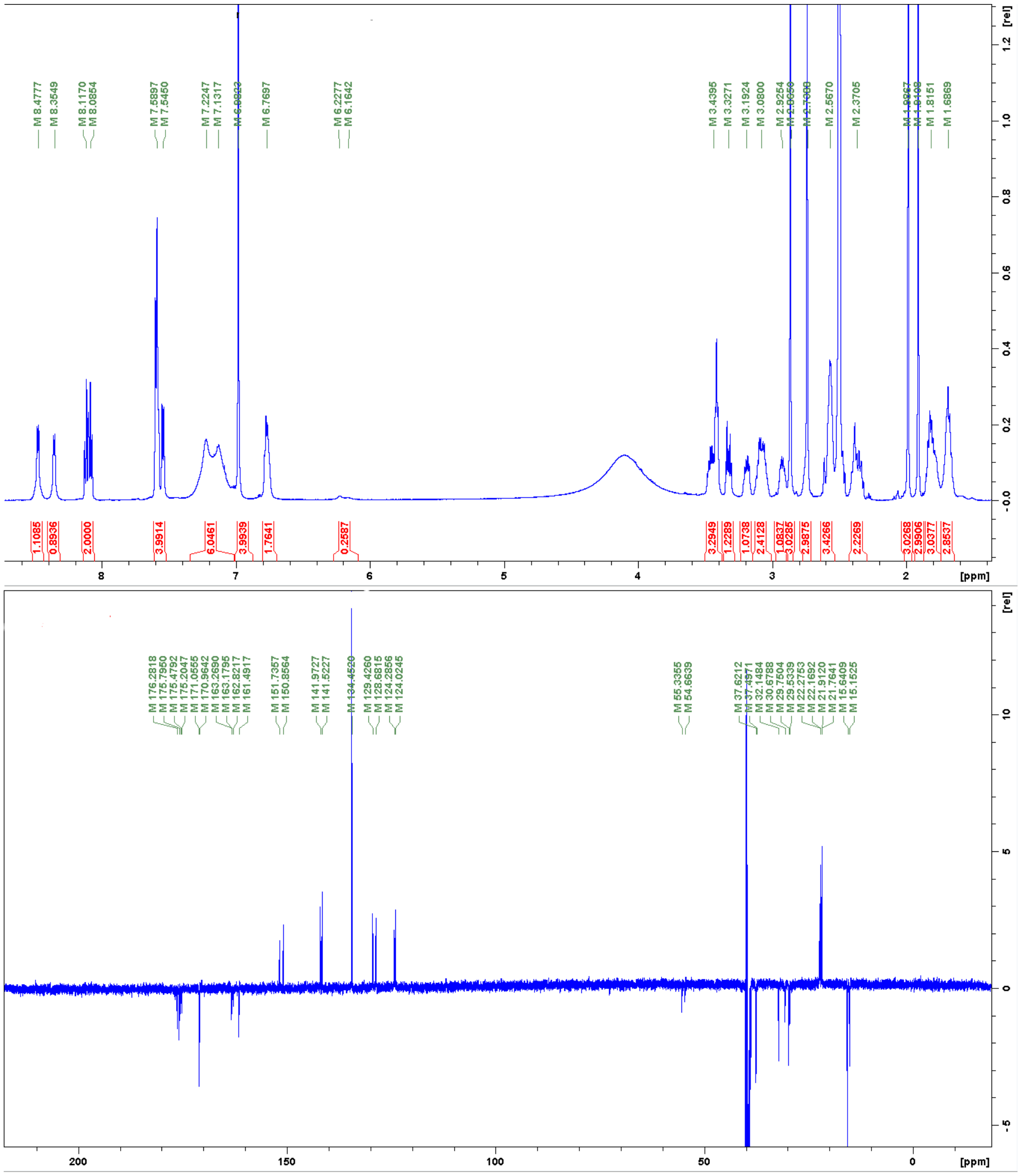

Figure S17. ${}^{1}H$ and ${}^{13}C$-NMR spectra of **PicoCarbo-Mal**, measured in DMSO-d6.

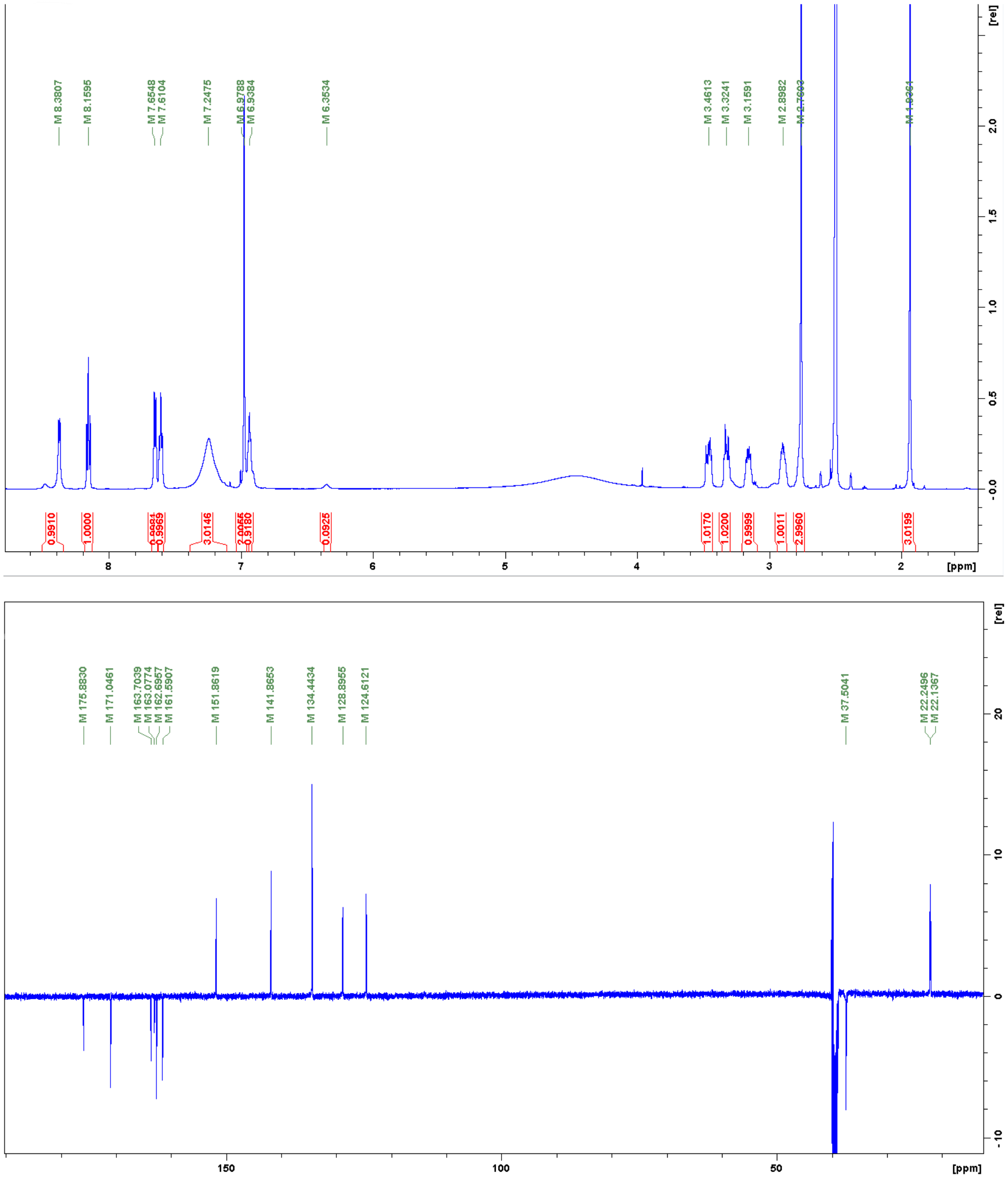

Figure S18. ^{1}H and ^{13}C-NMR spectra of **PicoOxali-Mal**, measured in DMSO-d6.
